# Edaphoclimatic Descriptors of Wild Tomato Species (*Solanum* Sect. Lycopersicon) and Closely Related Species (*Solanum* Sect. Juglandifolia and Sect. Lycopersicoides) in South America

**DOI:** 10.3389/fgene.2021.748979

**Published:** 2021-11-17

**Authors:** Gabriela Ramírez-Ojeda, Iris Edith Peralta, Eduardo Rodríguez-Guzmán, Jaime Sahagún-Castellanos, José Luis Chávez-Servia, Tulio Cecilio Medina-Hinostroza, Jorge Rodrigo Rijalba-Vela, Leopoldo Pompeyo Vásquez-Núñez, Juan Enrique Rodríguez-Pérez

**Affiliations:** ^1^ Crop Science Department, Horticulture Institute, Chapingo Autonomous University (UACh), Chapingo, Mexico; ^2^ Agronomy Department, Agricultural Sciences Faculty, National University of Cuyo (UNCUYO), Mendoza, Argentina; ^3^ Scientific Technological Center CONICET, Argentine Institute for Arid Zones Research, Mendoza, Argentina; ^4^ Agronomy Department, University Center for Biological and Agricultural Sciences, University of Guadalajara (UdG), Zapopan, Mexico; ^5^ Interdisciplinary Research Center for Integral Regional Development Oaxaca Unit, National Polytechnic Institute (IPN), Oaxaca, Mexico; ^6^ Directorate of Genetic Resources and Biosafety, Ministry of Environment, Lima, Peru; ^7^ Biological Sciences Faculty, National University Pedro Ruiz Gallo (UNPRG), Lambayeque, Peru

**Keywords:** wild tomatoes, edaphoclimatic diversity, ecological descriptors, genetic resources, canonical correlation analysis

## Abstract

Wild species related to cultivated tomato are essential genetic resources in breeding programs focused on food security to face future challenges. The ecogeographic analysis allows identifying the species adaptive ranges and most relevant environmental variables explaining their patterns of actual distribution. The objective of this research was to identify the diversity, ecological descriptors, and statistical relationship of 35 edaphoclimatic variables (20 climatic, 1 geographic and 14 edaphic variables) from 4,649 accessions of 12 wild tomato species and 4 closely related species classified in *Solanum* sect*.* Lycopersicon and clustered into four phylogenetic groups, namely “Lycopersicon group” (*S. pimpinellifolium*, *S. cheesmaniae*, and *S. galapagense*), “Arcanum group” (S*. arcanum*, *S. chmielewskii*, and *S. neorickii*), “Eriopersicon group” (*S. habrochaites*, *S. huaylasense*, *S. corneliomulleri*, *S. peruvianum*, and *S. chilense*), “Neolycopersicon group” (*S. pennellii*); and two phylogenetically related groups in *Solanum* sect. Juglandifolia (*S. juglandifolium* and *S. ochranthum*), and section Lycopersicoides (*S. lycopersicoides* and *S. sitiens*). The relationship between the climate and edaphic variables were determined by the canonical correlation analysis, reaching 89.2% of variation with the first three canonical correlations. The most significant climatic variables were related to humidity (annual evapotranspiration, annual precipitation, and precipitation of driest month) and physicochemical soil characteristics (bulk density, pH, and base saturation percentage). In all groups, ecological descriptors and diversity patterns were consistent with previous reports. Regarding edaphoclimatic diversity, 12 climate types and 17 soil units were identified among all species. This approach has promissory applications for biodiversity conservation and uses valuable genetic resources related to a leading crop.

## Introduction

Latin America and the Caribbean are regions rich in biodiversity, hosting nearly 60% of the world's biological diversity (UNEP-WCMC, 2016). Within this region, Mesoamerica is recognized as one of the main centers of origin, diversification, domestication, and biological plant diversity of various species of agricultural interest and animal consumption (Fortuny-Fernández et al., 2017). The complex evolutionary history, phylogenetics, geology, biogeography, and climatic variability are some factors that enhance the diversity in this area (UNEP-WCMC, 2016). This condition is essential to ensure food, socioeconomic, and cultural sovereignty for sustainable development and offers a large number of ecosystem services (FAO et al., 2019).

In this sense, tomato (*Solanum lycopersicum* L.) is one of the most cultivated vegetables due to its wide distribution and environmental adaptation in warm, subtropical, and tropical regions with nutritional and commercial importance worldwide (Peralta et al., 2008; Ramírez-Ojeda et al., 2021a). Regarding the place of origin and diversification of tomato, Peru is considered the center of origin with two transitions that involve tomato diversification process; the first one in South America, from wild species *S. pimpinellifolium* L. to a partially domesticated species *S. lycopersicum* L. var. *cerasiforme* (SLC); the second transition occurred in Mesoamerica from SLC to the completely domesticated species *S. lycopersicum* L. var. *lycopersicum*. However, new findings indicate that the origin of SLC may be prior to its domestication since many typical characteristics of tomatoes grown in South America come from this species; SLC is subsequently considered to have been lost or declined once the partially domesticated forms extended to the north (Razifard et al., 2020).

Wild species related to cultivated tomatoes are essential genetic resources in breeding programs focused on food security to face future challenges. Therefore, it is of strategic importance to study the climatic and edaphic factors that help to understand their current distribution patterns, as well as to establish the best indicators predicting possible effects of climate change and natural or anthropic environmental alterations. This is why it is necessary to undertake national and regional strategies for the conservation and use of cultivated and wild tomato genetic resources (Sandoval-Ceballos et al., 2021).

Based on an integrative taxonomy, which includes multiple evidences, the classification of wild tomatoes and their wild relatives was proposed: *Solanum* section Lycopersicon (Mill.) Wettst. comprises cultivated tomato (*S. lycopersicum* L.) and 12 wild tomato species: *S. arcanum* Peralta, *S. cheesmaniae* (L. Riley) Fosberg, *S. chilense* Dunal, *S. chmielewskii* (C. M. Rick, Kesicki, Fobes, and M. Holle), D. M. Spooner, G. J. Anderson, and R.K. Jansen, *S. corneliomulleri* J. F. Macbride, *S. galapagense* S. C. Darwin and Peralta, *S. habrochaites* S. Knapp and D. M. Spooner, *S. huaylasense* Peralta, *S. neorickii* D. M. Spooner, G. J. Anderson and R. K. Jansen, *S. pennellii* Correll, *S. peruvianum* L., and *S. pimpinellifolium* L. Four phylogenetically related *Solanum* species are also considered in the present study: *S. juglandifolium* Dunal, *S. ochranthum* Dunal (*Solanum* sect. Juglandifolia (Rydb.) A. Child), *S. lycopersicoides* Dunal, and *S. sitiens* I. M. Johnston (*Solanum* section *Lycopersicoides* (A. Child) Peralta) (Peralta et al., 2008; Causse et al., 2016; Tropicos.org, 2021).

Evidence of phylogenetic relationships of these species have been studied in detail by Peralta et al. (2008), who have proposed a six-group classification of wild tomatoes and phylogenetically closely related species: Section Lycopersicon: “Lycopersicon group” (*S. pimpinellifolium*, *S. cheesmaniae*, and *S. galapagense*), “Arcanum group” (*S. arcanum*, *S. chmielewskii*, and *S. neorickii*), “Eriopersicon group” (*S. habrochaites*, *S. huaylasense*, *S. corneliomulleri*, *S. peruvianum*, and *S. chilense*), “Neolycopersicon group” (*S. pennellii*); and two outgroups: Section Juglandifolia (*S. juglandifolium* and *S. ochranthum*) and Section Lycopersicoides (*S. lycopersicoides* and *S. sitiens*). This classification has been verified by molecular, genomic, and transcriptomic evidence of wild tomatoes (Rodríguez et al., 2009; Aflitos et al., 2014; Pease et al., 2016) and recently has been used for ecogeographic studies with satisfactory results (Ramírez-Ojeda et al., 2021a).

By considering plant genetic resources as the biological foundation for maintaining and improving crop productivity (Kantar et al., 2015), wild tomato species constitute an important gene pool due to the presence of genes with tolerance and resistance to biotic and abiotic factors (Arellano-Rodríguez et al., 2013; Cervantes-Moreno et al., 2014; Nosenko et al., 2016; Razali et al., 2018; Dinh et al., 2019) with potential use for breeding programs. Additionally, several questions arise about these gene pools, such as current distribution, population dynamics *in situ* or *ex situ*, and how are they used directly or as sources of genes to generate new varieties that respond to current and future basic problems of tomato cultivation (for example, climate change, diseases, pests), including the contribution of genes capable of conferring a greater nutritional–nutraceutical quality to new varieties (Chávez-Servia et al., 2011; Hernández-Bautista et al., 2014).

Identification of variables that derive in adaptation and speciation processes requires a large amount of field data of significant variables in natural populations. Recent developments and the use of remote sensing technologies, as well as a great availability of environmental information derived from Geographic information systems (GIS), have made it possible to identify patterns of species environmental variations at different scales (Nakazato et al., 2010). These tools and the availability of databases, with passport information of specimens collected in natural areas, allow for verification of the presence of species in a geographic range, as well as possible ecological descriptors, that is, to describe in detail the environmental conditions associated with the distribution of natural populations (Nakazato et al., 2010; Sánchez-González et al., 2018; Vilchez et al., 2019; Ministerio del Ambiente, 2020; Ramírez-Ojeda et al., 2021a).

One way to identify the adaptive ranges and most relevant variables that determine species distribution of valuable genetic resources is through ecogeographic studies, focusing on collection, conservation, characterization, documentation, and use of these resources (Parra-Quinajo et al., 2012; Pease et al., 2016), with the purpose of describing and explaining spatial patterns and processes involved in biodiversity distribution through time and space (Martiny et al., 2006; Tofalo et al., 2013; Délices et al., 2019). Ecogeographic studies of plant genetic resources allow the identification of the adaptive ranges of the species and the most relevant environmental variables that define their distribution (Parra-Quinajo et al., 2012; Ramírez-Ojeda et al., 2021a). Through ecogeographic studies, it is also possible to predict the environmental characteristics of the accession sites (Steiner and Greene, 1996) from ecological descriptors obtained through GIS tools using the geographical location and environmental variables (Lobo-Burle et al., 2013; Sánchez-González et al., 2018; Ramírez-Ojeda et al., 2021a, 2021b).

Currently, several information sources about geographical distribution of tomato species can be found in public databases (GBIF, 2021; Solanaceae Source, 2021; TGRC, 2021), conservation programs and gene banks (Córdoba-Téllez and Molia-Moreno, 2006; Florido et al., 2009; Magallanes-López et al., 2020), and genetic resources baseline studies (Ministerio del Ambiente, 2020), as well as some studies on geographic distribution patterns and ecological and climatic descriptors of wild tomato species (Peralta et al., 2008; Chetelat et al., 2009; Nakazato et al., 2010; Grandillo et al., 2011; Gonzá lez et al., 2013; Vilchez et al., 2019; Ramírez-Ojeda et al., 2021a). However, information regarding edaphic conditions of the sites where these species are located is limited or unknown (Balaguera-López et al., 2009).

Soil, a finite and nonrenewable natural resource, is of great importance in a large number of environmental services such as food and biomass production, climate regulation, carbon fixation, water storage and filtration, biogeochemical cycles, biodiversity reserve, and human physical and cultural environment (Burbano-Orjuela, 2016). Therefore, when considering edaphic together with climatic characteristics, it allows having a better understanding of the ecological and distribution patterns of the species.

Due to the limited edaphic information available regarding optimal characteristics for development of wild tomato species, the aim of the present work was to study ecological descriptors associated with soil characteristics and their relationship and the statistical association with climatic variables. Likewise, it was also analyzed whether the classification of wild tomatoes is related to the edaphoclimatic descriptors and supports the proposed groups of species.

## Materials and Methods

### Database

**FIGURE 1:**
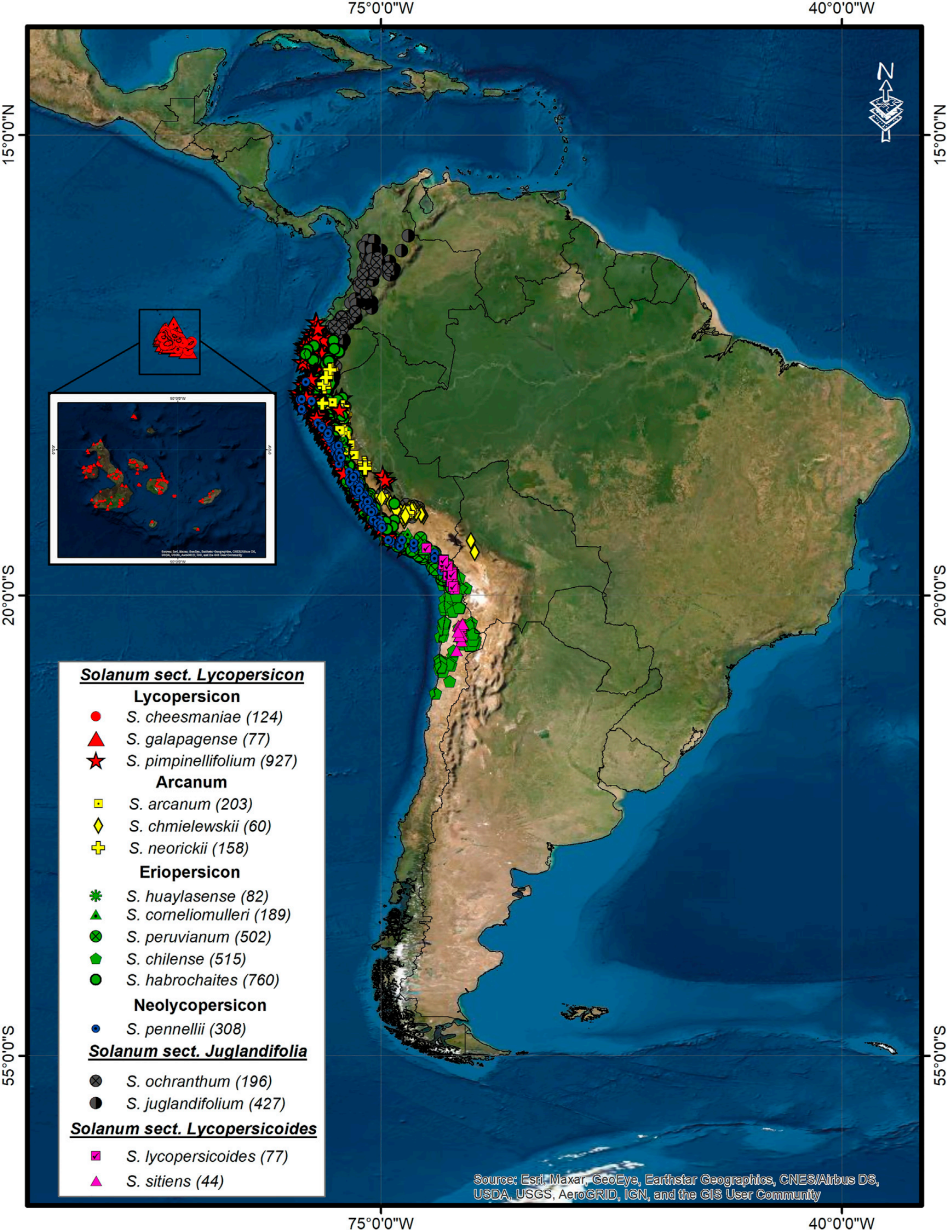
Geographic distribution of 12 wild tomato (*Solanum* sect. Lycopersicon) and 4 closely related species (*Solanum* sect. Juglandifolia, and Lycopersicoides). In parenthesis, number of accessions after each species name.

Initial database consisted of 12,131 accessions of 12 wild tomato species and 4 phylogenetically related species. Of these, 7,482 accessions were eliminated due to atypical data, repeated records, or accessions with little geographic precision and outside natural areas identified according to the altitude and ecological ranges reported (Peralta et al., 2008; Grandillo et al., 2011; Ministerio del Ambiente, 2020). The final 4,649 accessions database came from scientific reports, articles (Sotomayor et al., 2019; Razifard et al., 2020), international plant repositories (Tomato Genetic Resource Center, Global Biodiversity Information Facility, Solanaceae Source) (GBIF, 2021; Solanaceae Source, 2021; TGRC, 2021), and new accessions collected in 2018–2019 in Peru (Ministerio del Ambiente, 2020). The distribution of 16 species is shown in Figure 1. The species distribution is shown in Figure A1 in the Supplementary Material. It should be noted that *S. lycopersicum* was not included because its wide distribution would not reflect a natural but artificial distribution due to anthropic dispersal as a cultivated or ruderal species.

### Environmental Information

For the statistical analysis and ecological descriptors, an environmental information system with 900 m spatial resolution was built with 35 variables (Table 1). Nineteen bioclimatic variables were obtained from WorldClim version 2.1 from period 1970 to 2000 (Fick and Hijmans, 2017). Annual evapotranspiration (ET) was calculated from the sum of monthly values reported by Trabucco and Zomer (2019). Altitude (Alt), a geographic variable, was obtained with an elevation model from WorldClim (Fick and Hijmans, 2017). Alt was analyzed together with climatic variables due to the strong influence on the definition of climates. Finally, 14 edaphic variables obtained from the Harmonized World Soil Database version 1.1 (FAO/IIASA/ISRIC/ISSCAS/JRC, 2009) were used.

**TABLE 1:** Climatic, geographic, and edaphic variables used in the canonical correlation analysis and ecological descriptors.

Climatic variables
WorldClim variables (1970–2000): Annual mean temperature (**Bio1**, °C), mean diurnal range (**Bio2**, °C), isothermality (**Bio3**, Bio2/Bio7 × 100), temperature seasonality (**Bio4**, standard deviation × 100), maximum temperature of the warmest month (**Bio5**, °C), minimum temperature of coldest month (**Bio6**, °C), temperature annual range (**Bio7**, Bio5-Bio6), mean temperature of wettest quarter (**Bio8**, °C), mean temperature of driest quarter (**Bio9**, °C), mean temperature of warmest quarter (**Bio10**, °C), mean temperature mean of coldest quarter (**Bio11**, °C), annual precipitation (**Bio12**, mm), precipitation of wettest month (**Bio13**, mm), precipitation of driest month (**Bio14**, mm), precipitation seasonality (**Bio15**, coefficient of variation), precipitation of wettest quarter (**Bio16**, mm), precipitation of driest quarter (**Bio17**, mm), precipitation of warmest quarter (**Bio18**, mm), and precipitation of coldest quarter (**Bio19**, mm) (Fick and Hijmans, 2017). Annual evapotranspiration (**ET**, mm) (Trabucco and Zomer, 2019)
**Geographic variables**
Altitude (**Alt**, masl) (Fick and Hijmans, 2017)
**Edaphic variables**
Percentage of gravel (**GR**, %), sand (**SA**, %), silt (**SI**, %), and clay (**CL**, %), bulk density (**BD**, kg/dm^3^), organic carbon (**CO**, %), **pH**, cation exchange capacity (**CEC** cmol/kg), base saturation (**BS**, %), calcium carbonate (**CaCO_3_**, %), total exchangeable bases (**TEB**, cmol/kg), calcium sulfate (**CaSO_4_**, %), salinity (**SAL**, dS/m), and sodium (**SOD**, %) (FAO/IIASA/ISRIC/ISSCAS/JRC, 2009)

Edaphoclimatic diversity patterns were identified from climate types corresponding to world climatic classification proposed by Beck et al. (2018) with the Köppen–Geiger system and soil units from the Harmonized World Soil Database (FAO/IIASA/ISRIC/ISSCAS/JRC, 2009) (Table 2).

**TABLE 2:** Climate types and soil units used to determine edaphoclimatic diversity patterns.

Climate type
Af (tropical and rainforest), Am (tropical and monsoon), Aw (tropical and savannah), BWh (arid, desert, and hot), BWk (arid, desert, and cold), BSh (arid, steppe, and hot), BSk (arid, steppe, and cold), Csa (temperate, dry summer, and hot summer), Csb (temperate, dry summer, and warm summer), Csc (temperate and dry and cold summer), Cwa (temperate, dry winter, and hot summer), Cwb (temperate, dry winter, and warm summer), Cwc (temperate, dry winter, and cold summer), Cfa (temperate, no dry season, and hot summer), Cfb (temperate, no dry season, and warm summer), Cfc (temperate, no dry season, and cold summer), Dsa (cold, dry summer, and hot summer), Dsb (cold, dry summer, and warm summer), Dsc (cold, dry summer, and cold summer), Dsd (cold, dry summer, and very cold winter), Dwa (cold, dry winter, and hot summer), Dwb (cold, dry winter, and warm summer), Dwc (cold, dry winter, and cold summer), Dwd (cold, dry winter, and very cold winter), Dfa (cold, no dry season, and hot summer), Dfb (cold, no dry season, and warm summer), Dfc (cold, no dry season, and cold summer), Dfd (cold, no dry season, and very cold winter), ET (polar and tundra), and EF (polar and frost) (Beck et al., 2018)
**Soil units**
**AC** (Acrisol), **AL** (Alisol), **AN** (Andosol), **AR** (Arenosol), **AT** (Anthrosol), **CH** (Chernozem), **CL** (Calcisol), **CM** (Cambisol), **FL** (Fluvisol), **FR** (Ferralsol), **GL** (Gleysol), **GR** (Greysem), **GY** (Gypsisol), **HS** (Histosol), **KS** (Kastanozem), **LP** (Leptosol), **LV** (Luvisol), **LX** (Lixisol), **NT** (Nitisol), **PD** (Podzoluvisol), **PH** (Phaezem), **PL** (Planosol), **PT** (Plinthosol), **PZ** (Podzol), **RG** (Regosol), **SC** (Solonchak), **SN** (Solonetz), and **VR** (Vertisol) (FAO/IIASA/ISRIC/ISSCAS/JRC, 2009)

### Canonical Correlation Analysis and Ecological Descriptors

A selection of climatic and edaphic variables was made in order to identify a strong linear dependence (collinearity) between more than two explanatory variables. For this purpose, Pearson’s correlations were obtained, between variables, eliminating one of each pair whose absolute coefficient was greater than 0.90. The conserved variable was the one that showed the highest number of correlations with other variables, and therefore, the lowest number of non–linearly associated variables was maintained.

With the selected variables, a canonical correlation analysis was carried out to identify the relationship between the group of climatic variables and the group of edaphic variables. All statistical analyses were performed using SAS software (Statistical Analysis System) version 9.3 (SAS Institute, 2011).

Regarding ecological descriptors, these were calculated for each variable and each species (12 wild tomato and 4 phylogenetically related species) with the methodology proposed by Steiner and Greene (1996). Ecological descriptors were determined by vectors calculated with the geographic coordinates of each accession and the punctual value of each variable extracted with GIS.

Subsequently, the edaphic and climatic variables were identified as significant in the canonical correlation analysis; the extreme values (maximum and minimum), the median, and the coefficient of variation (CV = (Q/Med) × 100, where Q = (Q3 − Q1)/2 (interquartile range), and Med = median) were identified.

Finally, to identify the ecological distribution patterns of every group of species, altitude, annual mean temperature, precipitation, and annual evapotranspiration were considered as climatic variables and pH, cation exchange capacity (CEC), bulk density (BD), and base saturation (BS) as edaphic variables. These variables were chosen due to the importance and influence they have on the distribution and development of the species (Ramírez-Ojeda et al., 2021b), in addition to the importance and significance that they showed in the statistical analyses.

### Edaphoclimatic Diversity

**FIGURE 2:**
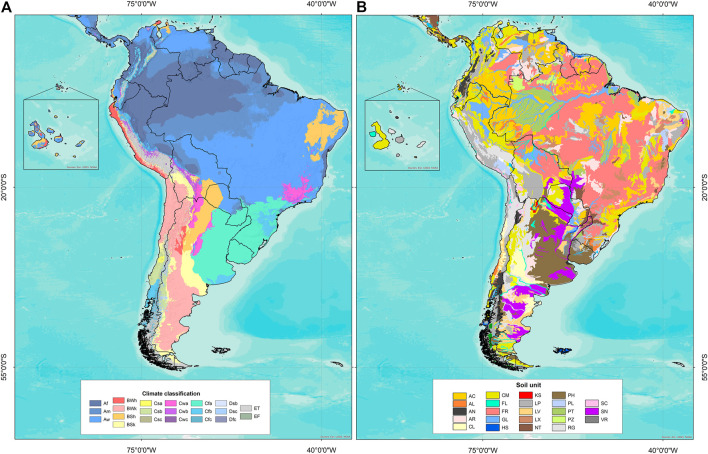
**(A)** Climate classification according to Beck et al. (2018): Af (tropical and rainforest), Am (tropical and monsoon), Aw (tropical and savannah), BWh (arid, desert, and hot), BWk (arid, desert, and cold), BSh (arid, steppe, and hot), BSk (arid, steppe, and cold), Csb (temperate, dry summer, and warm summer), Cwb (temperate, dry winter, and warm summer), Cfb (temperate, no dry season, and warm summer), and ET (polar, frost). **(B)** Soil units according to FAO/IIASA/ISRIC/ISSCAS/JRC (2009): AC (acrisol), AL (alisol), AN (andosol), AR (arenosol), CL (calcisol), CM (cambisol), FL (fluvisol), FR (ferralsol), GL (gleysol), HS (histosol), KS (kastanozem), LP (leptosol), LV (luvisol), LX (lixisol), NT (nitisol), PH (phaezem), PL (planosol), PT (plinthosol), PZ (podzol), RG (regosol), SC (solonchak), SN (solonetz), VR (vertisol).

Edaphoclimatic diversity was identified using GIS tools with the vector of geographic coordinates of each accession and raster images of climate types and soil units (Table 2). Figure 2 shows the distribution of climate types and soil units in South America. With the resulting information, a frequency table by climate type and soil unit was obtained for each species group (6) and for each individual species (16).

### Hot spot Analysis

Critical points of species abundance and areas with the greatest diversity concentration were established using ArcGIS with the “Spatial Statistics Tools” module. Spatial density maps were constructed by adding all those accessions of each species with a distance between accessions of 1 km. A distance criterion was chosen based on previous diversity studies of potato species (*Solanum* Sect. *Petota*), the sister group of tomatoes (Hijmans et al., 2002; Spooner et al., 2010). Subsequently, hot spot spatial analysis was performed with Getis-Ord Gi* statistic (Getis and Ord, 1992) to quantify the specific areas of high clustering and spatial significance for species abundance and diversity.

The hot spot analysis determines the spatial grouping of points higher (hot spot) or lower (cold spot) than the expected by a random distribution. Significance tests were calculated using z-values (Getis and Ord, 1992).

## Results

### Canonical Correlation Analysis and Ecological Descriptors

According to Pearson's correlation coefficients, out of the 34 edaphoclimatic variables, 19 did not present collinearity. The variables selected for subsequent statistical analyses and ecological descriptors were annual evapotranspiration, altitude, precipitation of dries month, annual precipitation, temperature annual range, isothermality, mean diurnal range, annual mean temperature, percentage of sand, silt and clay, BD, pH, organic carbon, CEC, BS, calcium carbonate CaCO_3_), sodicity, and salinity.

The canonical correlation analysis (CCA), performed with two groups of variables (climatic and edaphic), indicated that the first three canonical correlations had values of 0.800, 0.436, and 0.415, respectively, and percentages of explanation of data variation of 71.45, 9.38, and 8.36%, respectively, with a total of 89.20%. Likelihood ratio tests indicated that the three canonical correlations are different from zero (*p* ≤ 0.0001).

Correlations between climate characteristics and their canonical variables indicated that CLIMATE1 is associated with annual evapotranspiration (ET, −0.907), annual precipitation (Bio12, −0.947), and precipitation of driest month (Bio14, −0.864). The CLIMATE2 vector is associated with altitude (Alt, −0.5164) and isothermality (Bio3, −0.5642), which roughly quantifies the “hot” (low values) and “cold” (high values) regions. The CLIMATE3 vector represents the mean annual temperature (Bio1, 0.7145) and mean diurnal range (Bio2, 0.793).

Regarding correlations of soil canonical variables, the SOIL1 vector represent BD (0.801), pH (0.658), and BS percentage (BS, 0.707). High values of the SOIL2 vector identify soils with high content of sand (0.478), pH (0.552), CaCO_3_ concentration (0.546), and low clay content (−0.427). Finally, the SOIL3 vector does not show important correlations.

The correlations between the CLIMATE vectors with the original soil variables indicate that CLIMATE1 had correlations of importance with BD (0.641), pH (0.527), CEC (−0.452), BS (0.566), and CaCO_3_ content (0.424). CLIMA2 and CLIMA3 did not show correlation of importance. SOIL1 with climate variables showed associations with annual evapotranspiration (ET, −0.7267), annual precipitation (Bio12, −0.7585), precipitation of driest month (Bio14, −0.6924), isothermality (Bio3, 0.5404), and temperature annual range (Bio7, 0.5117). SOIL2 and SOIL3 did not show correlation of importance.

**FIGURE 3:**
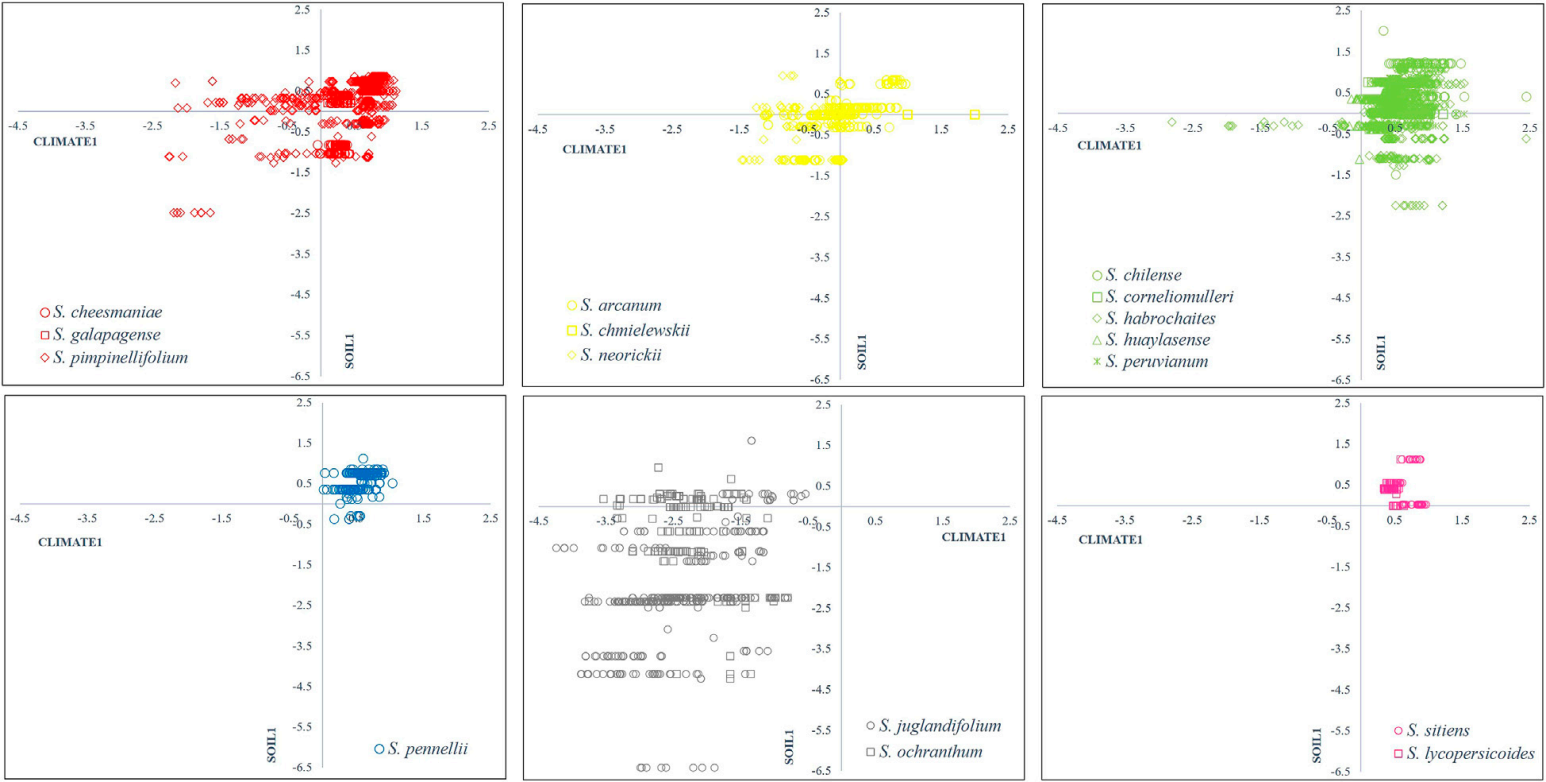
Scatter plot of SOIL1 vs CLIMATE1 canonical vectors from CCA with 0.80 correlation and 74% of total variation. Group 1 (red): Section Lycopersicon: “Lycopersicon group”, Group 2 (yellow): “Arcanum group”, Group 3 (green): “Eriopersicon group”, Group 4 (blue): “Neolycopersicon group” outgroup close related species in Section Juglandifolia Group 5 (grey): and Group 6 (pink): Section Lycopersicoides. In each scatter plot first-quadrant (I) represents environments with low annual evapotranspiration (ET), annual precipitation (Bio12), precipitation of driest month (Bio14) and high pH, BD, and base saturation (BS); second-quadrant (II) represents environments with high ET, Bio12, Bio14 and high pH, BD and BS; third-quadrant (III) represents environments with high ET, Bio12, Bio14 and low pH, BD and BS; fourth-quadrant (IV) represents environments with low ET, Bio12, Bio14, and low pH, BD, and BS.

Figure 3 shows the relationship between canonical variables CLIMATE1 and SOIL1, representing 71.45% of the total data variability and a positive correlation of both canonical variables of 0.80. This figure shows the distribution and ecological adaptation of every species regarding canonical correlations.

Table 3 and Table 4 show the ecological descriptors of edaphic and climatic variables identified as significant in the first and second canonical correlation. These results are mostly consistent with the environmental ranges previously reported in other studies. Table A1 in Supplementary Material shows the ecological descriptors of the rest of the variables.

**TABLE 3:** Ecological descriptors of climatic and edaphic variables associated with the first canonical correlation (71.4%) for12 species of wild tomato and 4 closely related species. Bio12 = annual precipitation, Bio14 = precipitation of driest month, pH = hydrogen ion concentration. ^*^Range (maximum–minimum value), ^**^median, ^***^coefficient of variation.

Group/Section	Species	Bio12 (mm)	Bio14 (mm)	ET (mm)	BD (kg/dm^3^)	pH	BS (%)
Lycopersicon	*S. cheesmaniae*	107–562^*^	0–15	187–1,125	1.1–1.4	4.3–8.5	31.0–100
		277^**^ (21.3)^***^	3 (66.6)	45 (41.9)	1.3 (1.1)	7.1 (15.4)	100 (34.5)
	*S. galapagense*	135–546	0–15	160–1,052	1.1–1.4	4.3–8.5	31.0–100
		274 (16.0)	4 (50)	531 (30.6)	1.3 (0.4)	4.9 (22.4)	32.0 (107.8)
	*S. pimpinellifolium*	1–2,828	0–143	1–1,710	0.3–1.5	3.2–8.5	10.0–100
		68 (146.3)	0 (0)	43 (195.9)	1.4 (6.5)	7.6 (9.2)	100 (4.5)
Arcanum	*S. arcanum*	22–1,193	0–55	11–1,094	0.3–1.5	4.6–8.5	17.0–100
		487 (44.0)	4 (137.5)	390 (43.2)	1.3 (1.5)	6.4 (13.2)	87.0 (5.1)
	*S. chmielewskii*	504–1,318	4–19	429–874	1.2–1.5	5.2–8.1	19.0–100
		944 (18.2)	11 (22.7)	610 (16.9)	1.2 (2.5)	8.1 (4.3)	100 (0)
	*S. neorickii*	426–1,366	3–68	326–1,031	0.2–1.7	4.6–8.1	17.0–100
		817 (21.2)	18.5 (45.9)	672 (13.7)	1.2 (10.2)	5.6 (26.7)	81 (44.0)
Eriopersicon	*S. huaylasense*	128–507	0–3	73–424	1.2–1.4	5.2–7.9	21.0–100
		328 (21.5)	1 (50)	238 (23.0)	1.4 (7.9)	5.6 (12.5)	38.0 (81.5)
	*S. corneliomulleri*	19–434	0–2	12–354	1.1–1.5	4.2–8.1	19.0–100
		205 (59.2)	0 (0)	141 (45.7)	1.4 (6.7)	5.6 (16.9)	38.0 (86.8)
	*S. peruvianum*	0–534	0–3	0–427	1.2–1.5	3.2–8.6	10.0–100
		25 (97.4)	0 (0)	13 (134.6)	1.3 (4.1)	7.6 (6.3)	100 (5.0)
	*S. chilense*	0–355	0–1	3–275	1.0–1.5	4.2–8.6	28.0–100
		29 (68.9)	0 (0)	20 (72.5)	1.3 (2.3)	7.5 (10.6)	100 (6)
	*S. habrochaites*	11–2,358	0–143	8–1,682	0.3–1.7	4.3–8.5	14.0–100
		605 (42.0)	3 (266.6)	535 (43.9)	1.3 (7.6)	5.7 (15.7)	87 (35.6)
Neolycopersicon	*S. pennellii*	1–404	0–3	0–289	1.2–1.5	5.1–8.5	19.0–100
		49 (94.8)	0 (0)	33 (95.8)	1.3 (4.9)	7.9 (14.5)	100 (31.0)
Juglandifolia	*S. juglandifolium*	555–3,214	1–194	413–1,648	0.3–1.9	4.1–7.7	13.0–100
		1,895 (28.7)	60 (49.1)	1,177 (10.0)	0.9 (1.0)	5.2 (1.9)	23.0 (36.9)
	*S. ochranthum*	507–2,358	2–131	387–1,474	0.3–1.7	3.2–8.5	10.0–100
		1,010 (11.0)	36 (43)	814 (14.1)	1.2 (11.2)	5.6 (13.3)	45.0 (67.7)
Lycopersicoides	*S. lycopersicoides*	13–215	0–0	9–182	1.3–1.4	4.7–8.1	34.0–100
		104 (50.4)	0 (0)	82 (58.5)	1.3 (1.5)	7.5 (10.6)	100 (6.5)
	S. sitiens	8–31	0–0	9–26	1.2–1.4	6.4–7.9	88.0–100
		17 (24.2)	0 (0)	21 (16.6)	1.2 (8.5)	6.5 (10.7)	88.0 (6.8)

**TABLE 4:** Ecological descriptors of climatic and edaphic variables associated with the second canonical correlation (9.3%) for 12 species of wild tomato and 4 closely related species. Bio3 = isothermality, Sand = sand percentage, Clay = clay percentage. ^*^Range (maximum–minimum value), ^**^median, ^***^coefficient of variation.

Group/Section	Species	Alt (m)	Bio3 (°C × 100)	Sand (%)<	CaCO_3_ (%)	Clay (%)
Lycopersicon	*S. cheesmaniae*	5–1,478^*^	58.3–74.8	33–60	0–3.1	3–37
		87^**^ (152.4)^***^	65 (4.5)	43 (11.6)	2 (67.3)	28 (16.0)
	*S. galapagense*	4–868	59.1–73.9	33–60	0–3.1	3–37
		45 (240.0)	68.3 (4.9)	34 (14.7)	0 (0)	36 (12.5)
	*S. pimpinellifolium*	1–1,774	48.2–89.4	0–94	0–4.3	0–56
		92 (101.9)	65.6 (8.0)	44 (51.1)	2 (65)	17 (47)
Arcanum	*S. arcanum*	132–3,292	65.5–90.1	0–83	0–4.3	0–45
		1,767 (33.1)	87 (2.7)	54 (10.1)	0 (0)	17 (8.8)
	*S. chmielewskii*	1,803–3,195	73.6–86.6	25–63	0–2.0	14–31
		2,445 (11.3)	82.6 (2.9)	63 (12.6)	2 (17.5)	14 (14.2)
	*S. neorickii*	1,202–3,262	76.4–89.1	0–76	0–2.4	0–56
		2,230 (12.2)	84.5 (1.9)	63 (30.1)	0 (0)	12 (20.8)
Eriopersicon	*S. huaylasense*	978–3,304	80.7–90.2	25–67	0–3.5	10–28
		2,301 (18.8)	87.8 (1.1)	67 (31.3)	0 (0)	10 (85)
	*S. corneliomulleri*	1,018–3,097	64.6–87.4	25–80	0–4.0	4–32
		2,310 (17.0)	75.8 (3.9)	57 (16.6)	0 (0)	16 (43.7)
	*S. peruvianum*	2–3,191	40.5–87.8	25–94	0–21.6	2–32
		532, (128.0)	62.5 (15.3)	54 (21.2)	2.9 (55.1)	16 (31.2)
	*S. chilense*	0–3,995	41.6–87.2	30–96	0–21.6	1–32
		1,910 (57.9)	68.1 (10.1)	54 (24.0)	3.1 (59.6)	19 (31.5)
	*S. habrochaites*	40–3,692	50.1–91.0	0–94	0–4.3	0–46
		2,137 (30.6)	84.5 (5.1)	51 (32.3)	0 (0)	17 (44.1)
Neolycopersicon	*S. pennellii*	5–2,921	48.0–87.4	25–94	0–7.2	2–28
		831 (52.4)	68.5 (8.2)	54 (12)	2.9 (60.3)	16 (18.7)
Juglandifolia	*S. juglandifolium*	1,005–3,153	76.5–94.5	9–94	0–2.0	2–56
		2,195 (14.3)	89.2 (1.9)	40 (25)	0 (0)	13 (46.1)
	*S. ochranthum*	1,195–4,008	72.4–93.8	0–83	0–2.4	0–46
		2,742 (10.5)	85.8 (3.3)	60 (16.6)	0 (0)	12 (26.0)
Lycopersicoides	*S. lycopersicoides*	1,290–3,775	65.8–85.5	30–57	0–7.2	18–32
		2,960 (13.2)	73.1 (4.6)	50 (11)	3.1 (53.2)	20 (20)
	*S. sitiens*	2,276–3,330	67.9–71.6	43–69	0.3–7.2	12–28
		2,740 (5.7)	69.1 (1.2)	69 (8.6)	0.3 (1,150)	12 (25)

**FIGURE 4:**
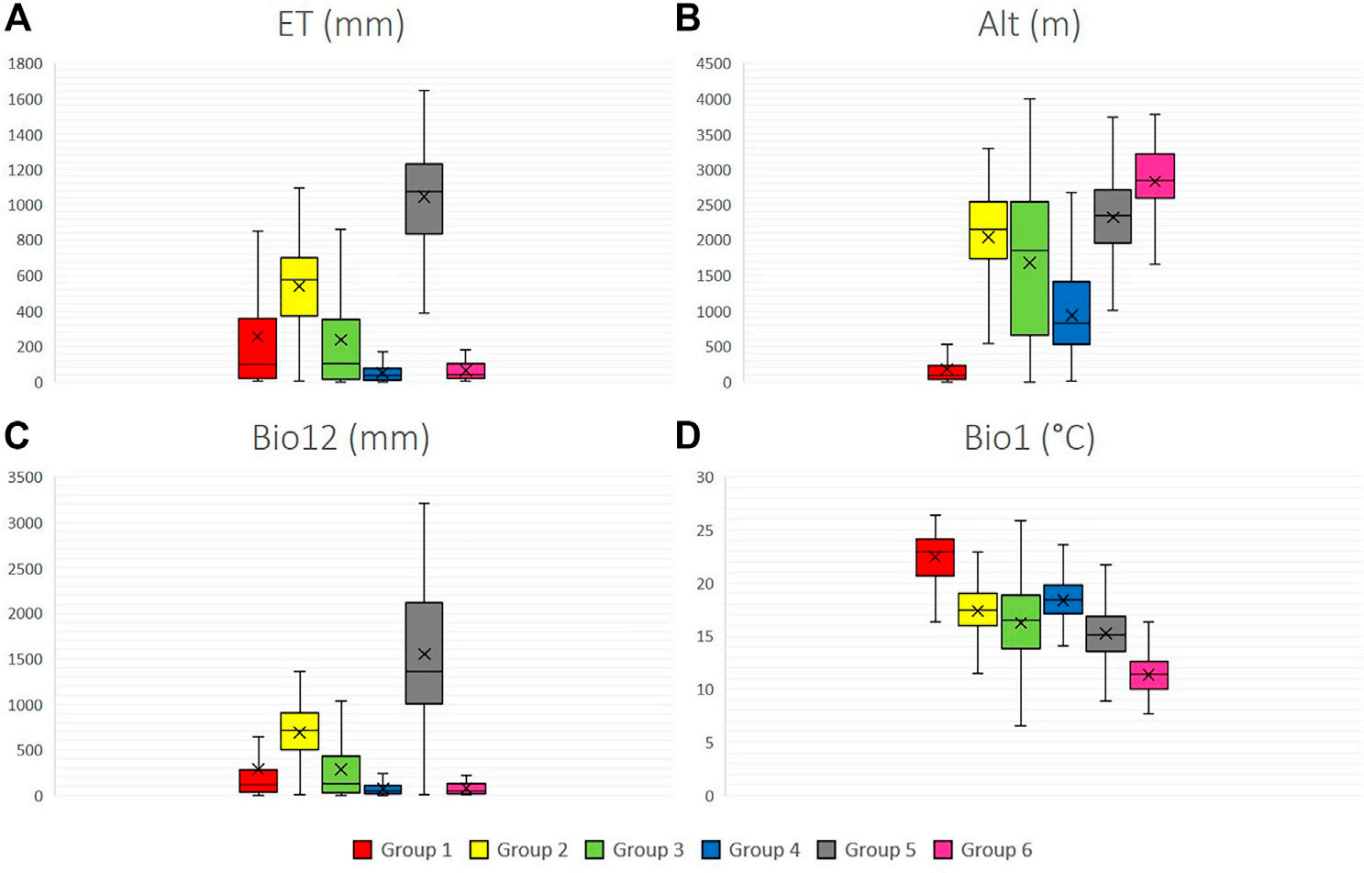
Climate variables boxplots for six groups of 12 wild tomato species and 4 closely related species. **(A)** ET (annual evapotranspiration), **(B)** Alt (altitude), **(C)** Bio12 (annual precipitation), **(D)** Bio1 (mean annual temperature). Group 1: Section Lycopersicon: “Lycopersicon group” (*S. pimpinellifolium*, *S. cheesmaniae*, and *S. galapagense*), Group 2: “Arcanum group” (*S. arcanum*, *S. chmielewskii*, and *S. neorickii*), Group 3: “Eriopersicon group” (*S. habrochaites*, *S. huaylasense*, *S. corneliomulleri*, *S. peruvianum*, and *S. chilense*), Group 4: “Neolycopersicon group” (*S. pennellii*); outgroup close related species in section Juglandifolia Group five (*S. juglandifolium* and *S. ochranthum*) and Group six: Section Lycopersicoides (*S. lycopersicoides* and *S. sitiens*).

Figure 4 shows the boxplots for four climatic variables for each of the six species groups, as well as the amplitude observed for each variable.

Among the main findings, it can be observed that groups 4 (*S. pennellii*) and 6 (*S. lycopersicoides* and *S. sitiens*) are ones that contain the species that distributes in environments with the lowest availability of precipitation and evapotranspiration. Considering altitude, group 1 (*S. pimpinellifolium*, *S. cheesmaniae*, and *S. galapagense*) has the lowest average altitude, while group 6 (*S. lycopersicoides* and *S. sitiens*) has the highest average altitude. Group 1 was located in environments with the highest mean annual temperature; by contrast, group 6 had the lowest average annual temperature. Groups 2, 3, and 5 remained in transition climatic conditions with the rest of the phylogenetic groups.

**FIGURE 5:**
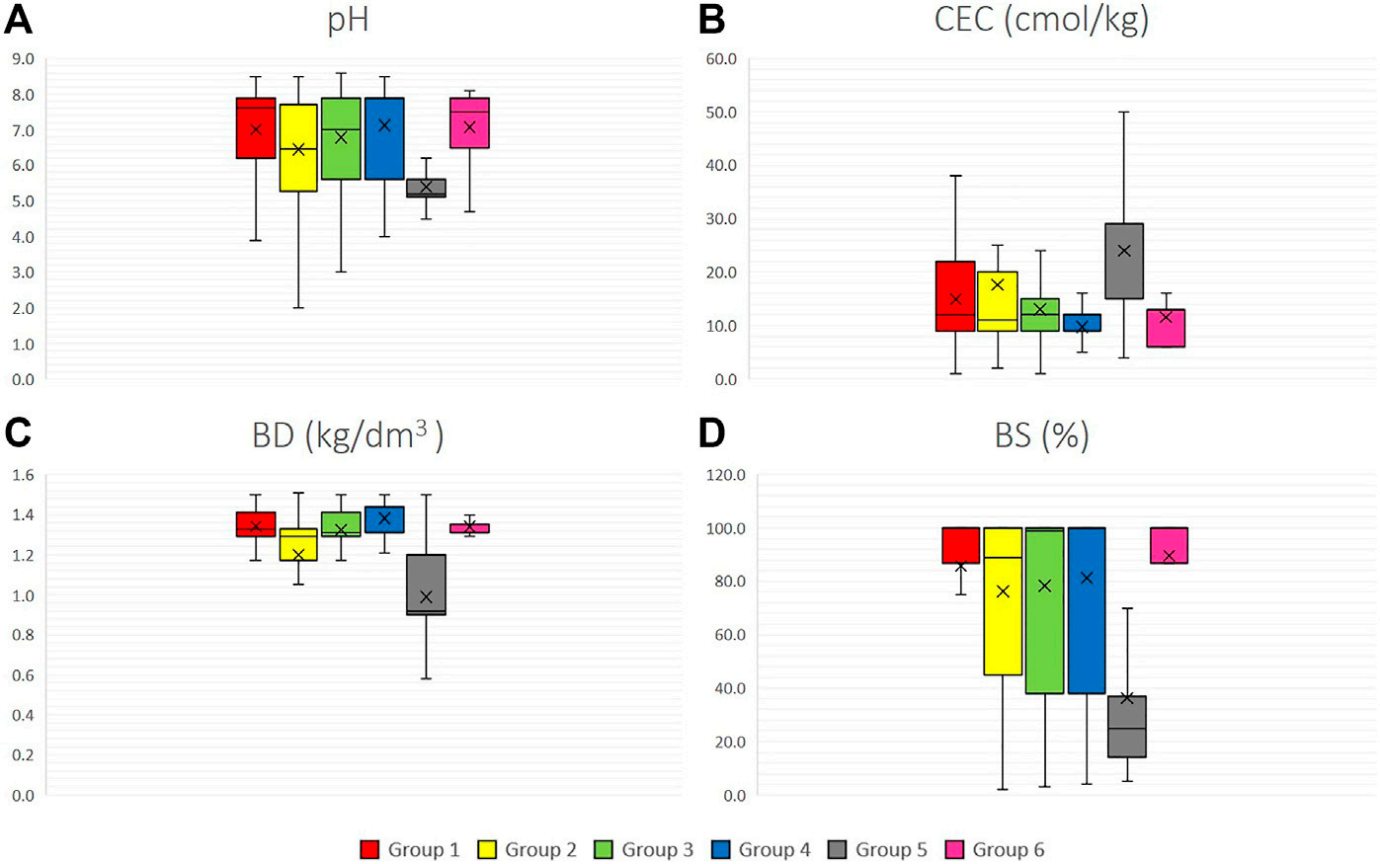
Edaphic variables boxplots for six groups of the 12 wild tomato species and 4 related species **(A)** pH, **(B)** CEC (cation exchange capacity), **(C)** BD (bulk density), **(D)** BS (base saturation). Group 1: Section Lycopersicon: “Lycopersicon group” (*S. pimpinellifolium, S. cheesmaniae* and *S. galapagense*), Group 2: “Arcanum group” (*S. arcanum*, *S. chmielewskii*, and *S. neorickii*), Group 3: “Eriopersicon group” (*S. habrochaites*, *S. huaylasense*, *S. corneliomulleri*, *S. peruvianum*, and *S. chilense*), Group 4: “Neolycopersicon group” (*S. pennellii*); outgroup close related species in Section Juglandifolia Group 5 (*S. juglandifolium* and *S. ochranthum*) and Group 6: Section Lycopersicoides (*S. lycopersicoides* and *S. sitiens*).

The analysis of four edaphic variables in Figure 5 determines that group 5 (*S. juglandifolium* and *S. ochranthum*) has the lowest pH average. In all groups, BD was relatively constant, with similar values in all species. The mean BS in most of the groups was greater than 80%, except for group 5, with an average value around 40%. In general, soil characteristics in all groups of species were relatively similar, except for group 5 (*S. ochranthum* and *S. juglandifolium*) which presented an opposite trend.

### Edaphoclimatic Diversity

**FIGURE 6:**
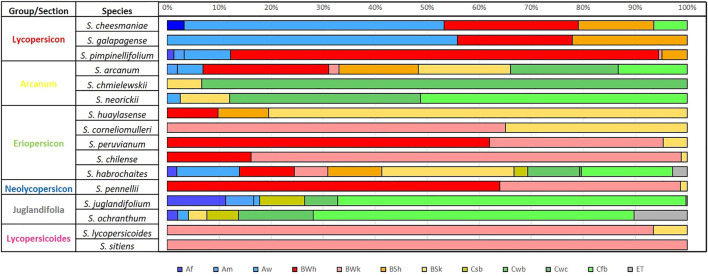
Percentage of climate type by species according to Beck et al. (2018) of 12 wild tomato (*Solanum* Sect. Lycopersicon) and 4 closely related species (*Solanum* Sect. Juglandifolia and Sect. Lycopersicoides). Climate type: Af (tropical, rainforest), Am (tropical, monsoon), Aw (tropical, savannah), BWh (arid, desert, hot), BWk (arid, desert, cold), BSh (arid, steppe, hot), BSk (arid, steppe, cold), Csb (temperate, dry summer, and warm summer), Cwb (temperate, dry winter, and warm summer), Cfb (temperate, no dry season, and warm summer), ET (polar, frost).

**FIGURE 7:**
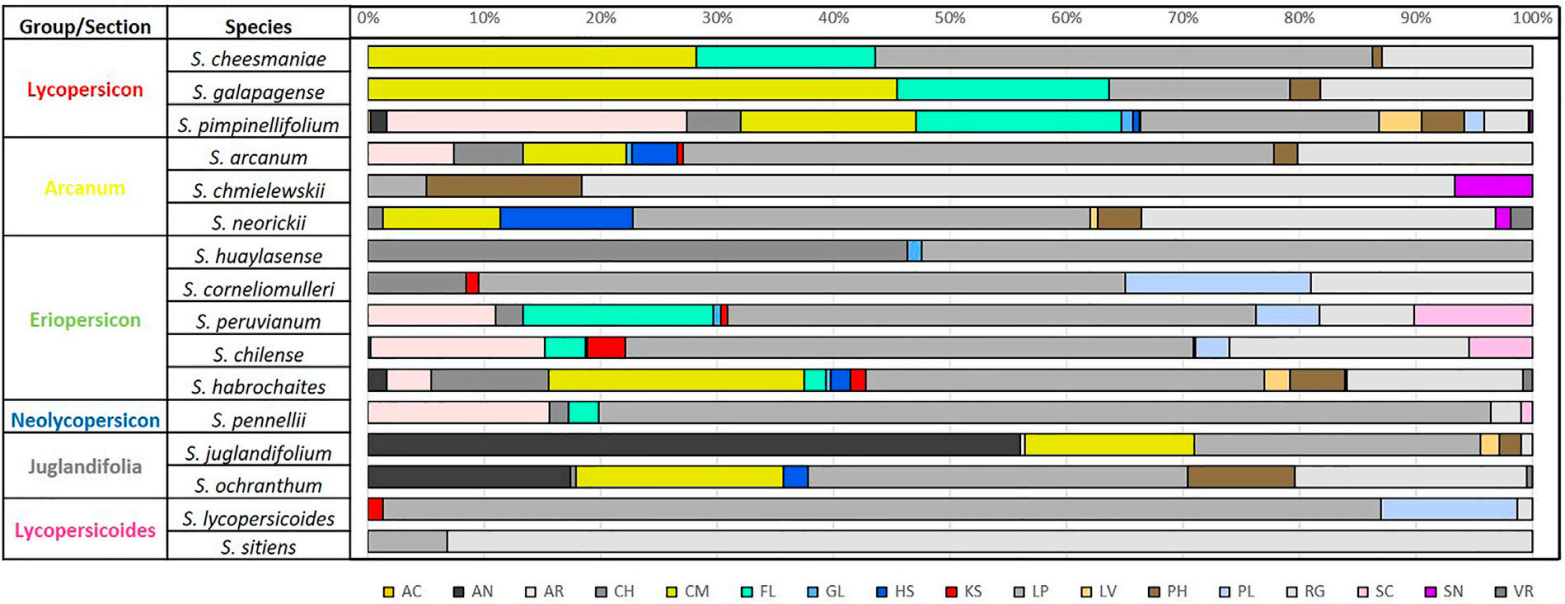
Percentage of soil type by species according to FAO/IIASA/ISRIC/ISSCAS and JRC (2009), for 12 wild tomato (*Solanum* sect. Lycopersicon) and 4 closely related species (*Solanum* sect. Juglandifolia and sect. Lycopersicoides). Soil type: AC (acrisol), AN (andosol), AR (arenosol), CH (chernozem), CM (cambisol), FL (fluvisol), GL (gleysol), HS (histosol), KS (kastanozem), LP (leptosol), LV (luvisol), PH (phaezem), PL (planosol), RG (regosol), SC (solonchak), SN (solonetz), and VR (vertisol).

The edaphoclimatic diversity found in 16 species is shown in Figure 6 and Figure 7. Regarding, climate diversity, it was possible to identify 12 climate types of the 21 reported for Latin America by Beck et al. (2018).

Within the six phylogenetically related groups identified by Peralta et al. (2008) and used by Ramírez-Ojeda et al. (2021a), specific climate type patterns can be observed, with the same climate types occurring in different proportions within each group (Figure 6), confirming in most of the groups, the environmental distribution similarity between the species that make them up.

*S. habrochaites* has the greatest diversity (11 climate types), intermedium diversity (8 climate types) was found in *S. arcanum*, while *S. sitiens* has the greatest climatic restriction, located only in climates BWk (arid, desert, cold). The climate type identified in most of the accession sites was associated with the 16 species was BSk (arid, steppe, and cold), and only absent in species of Lycopersicon group (S*. cheesmaniae*, *S. galapagense*, and *S. pimpinellifolium*) and in *S. juglandifolium* and *S. sitiens*. The opposite case was presented with Cwc climate (temperate, dry winter, and cold summer) present only in some areas where *S. habrochaites* was collected. *S. juglandifolium* and *S. ochranthum* share similar climatic types but were most frequently found in Cfb (temperate, no dry season, warm summer).

Diversity of soil units among wild tomato species (Figure 7) found 17 different soil units out of the 23 reported for Latin America in Harmonized World Soil Database from FAO/IIASA/ISRIC/ISSCAS and JRC (2009). Most frequent soils types were leptosol (LP), present in all species, regosol (except *S. huaylasense*) and less frequently acrisol (AC), present only in some *S. pimpinellifolium* accessions.

The greatest edaphic diversity was found in *S. pimpinellifolium*, with accessions in 16 of the 17 reported soil units (except VR). The opposite case was identified for species of Lycopersicoides section *S*. *sitiens* and *S. lycopersicoides*, with two and four soil units, respectively. Likewise in the patterns of climatic diversity described, edaphic diversity is similar within species, integrating each of the six phylogenetically related groups.

### Hot spot Analysis

**FIGURE 8:**
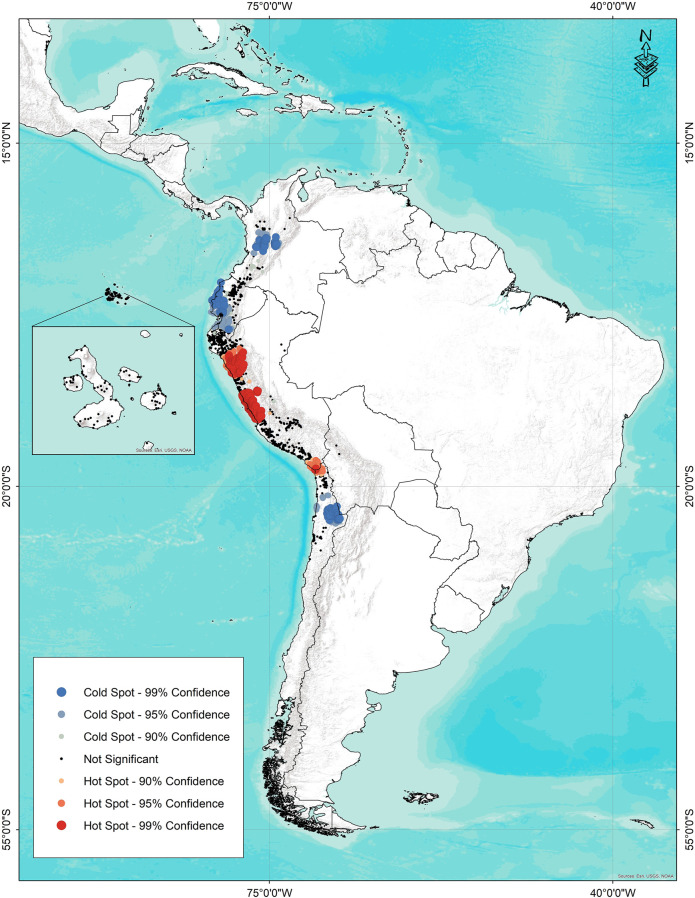
Species diversity map hot spots (red) and cold spots (blue) for 12 wild tomato (*Solanum* Sect. Lycopersicon) and 4 closely related species (*Solanum* sect. Juglandifolia and sect. Lycopersicoides).

Areas with a high number of species and accessions were determined by hot spot analysis. Figure 8 shows the result of hot spot analysis applied with a distance of 1 km between accessions for 4,649 accessions of 12 wild tomato and 4 phylogenetically related species. The highest concentration of species is located in two areas of Peru, one near Trujillo and Chimbote, and the second area around Lima. Likewise, a small area with high diversity is located in southern Peru and northern limit of Chile. The zone in Trujillo–Chimbote is characterized by the presence of seven species (*S. pennellii*, *S. arcanum*, *S. neorickii*, *S. huaylasense*, *S. habrochaites*, *S. pimpinellifolium*, and *S. ochranthum*). The region of high diversity around *Lima* also features seven species: *S. pennellii*, *S. neorickii*, *S. corneliomulleri*, *S. peruvianum*, *S. chilense*, *S. habrochaites*, and *S. pimpinellifolium*.

Finally, the region of high diversity on the border between Chile and Peru is home to five species: *S. pennellii*, *S. peruvianum*, *S. chilense*, *S. pimpinellifolium*, and *S. lycopersicoides*.

Cold spots correspond to the geographical distribution of *S. ochranthum* and *S. juglandifolium* accessions in Colombia and Ecuador, and *S. sitiens* in the northern region of Chile. The rest of the areas of distribution are insignificant according to the statistical criteria, assuming a random distribution.

## Discussion

This research provides a relevant ecogeographic characterization to understand the distribution patterns of wild species that complement the phenotypic and genetic information. Characterization of genetic resources through environmental characteristics associated with accession areas and use of GIS tools allows the identification of adaptive ranges and most relevant environmental factors affecting species distribution and ecological adaptation (Parra-Quinajo et al., 2012).

Likewise, through GIS and georeferenced information of species locations, it is possible to quantify geographical distances and distribution patterns of germplasm accession sites. From this perspective, it is likely to determine specific environmental conditions in which wild species and local varieties of crops have acquired their adaptive characters (Hijmans and Spooner, 2001). Therefore, the results obtained in this research constitute a source of updated and valuable information on the edaphoclimatic characteristics in which wild tomatoes and phylogenetically related species are distributed along its natural geographic range.

In general, geographical distribution of 16 wild species related to the cultivated tomato is wide, from Colombia through Peru, comprising Pacific coastal region to Chile and the Andean mountains, with an altitudinal range from sea level to 3,300 m (Peralta et al., 2008; Bergougnoux, 2014. However, within this distribution, there are overlapping areas between several species or regions with specific distribution such as the endemic species of the Galapagos Islands (*S. cheesmaniae* and *S. galapagense*) or hyper arid regions of northern Chile with other rare endemic species, *S. sitiens*. Within these distribution patterns, it is also possible to identify differences and similarities between the species that conform each group, for example, the similarity between *S. arcanum* and the species of Lycopersicon group (Figure 6), reflecting a wider distribution and adaptations to local sites of ecotypes (Peralta et al., 2008). These environmental characteristics reflect the ecological adaptation patterns and habitat preference of each species (Nakazato et al., 2010; Vilchez et al., 2019) (Figures 6, 7, Table A1 and A3 in Supplementary Material). It is worth mentioning that these results also suggest a thorough revision of the proposed groups, incorporating the new passport data as well as genetic and molecular information to corroborate the belonging of each species to the phylogenetic assigned groups. The aforementioned are under the assumption that the species are closely and genetically related and in expecting that their adaptation areas are similar.

Regarding wild tomato species and phylogenetically related species, few studies have been carried out with an ecogeographic or climatic focus. A comprehensive treatment integrates main botanical, biological, and ecological characteristics of each wild tomato and related species (Peralta et al., 2008); other studies focused on distribution of species richness and diversity through the analysis with GIS (González, 2013) and established conservation priorities (Vilchez et al., 2019); further geographical and ecological characterization have been investigated in 10 tomato species determining soil and climate variables (Nakazato et al., 2010); studies have been conducted on tomato biogeography, *S. lycopersicum* var. *cerasiforme*, in its center of origin and domestication (Délices et al. (2019); and finally climatic effects on species distribution (Lin et al., 2020) and bioclimatic characterization, and identification of ecological descriptors and patterns of climatic diversity of 12 wild tomato and 4 closely related species (Ramírez-Ojeda et al., 2021a) have been studied. In this sense, this study complements the information available, providing information on soil characteristics that had not been analyzed.

The canonical correlation analysis satisfactorily identifies climatic variables with greatest influence on edaphic variables and vice versa, with a correlation of 0.80 representing 74% of total variation in 4,649 accessions. One main conclusion is that variables related to water availability (ET, Bio12, Bio14) have a great influence on physical (BD) and chemical soil characteristics (BS, pH). This pattern is persistent in all six groups. This relationship can be better observed in group 5 (*S. juglandifolium* and *S. ochranthum*) accessions with greater availability of annual precipitation and evapotranspiration, which present lower pH, BD, and BS than the rest of species; that is, they are located in soils with the lowest agricultural quality (Figures 4, 5). This methodological approach is promising to be applied at other scales, considering the analysis at population level of each species and climatic and edaphic factors limited to smaller areas of distribution. This basis of ecogeographic characterization could incorporate information from genetic and ecological studies. A better understanding of these variables would allow the generation of projection models in different climate change scenarios (Violle and Jiang, 2009;Luebert and Weigend, 2014; Godoy-Bürki, 2016; Lin et al., 2020).

Ecological descriptors obtained, despite the incorporation of new accessions, are very similar to the ranges reported by Peralta et al. (2008) and Ramírez-Ojeda et al. (2021a) and generally identify the groups of species proposed in the classification. It is important to mention that this methodology has been widely used in the study of other species (Ruiz-Corral et al., 2008; Cerda-Hurtado et al., 2018; Sánchez-González et al., 2018; Ramírez-Ojeda et al., 2021a; 2021b). With this information, it is also possible to identify those species with tolerance to extreme conditions, for example, low and high temperatures, humidity conditions, altitude, pH, BD, and all the possible conditions when associating a species with a climate type or soil unit (Table 3 and Table 4, Tables A1, A2, and A3 in Supplementary Material).

Edaphic diversity (Figure 7) tends to be more constant between species groups and sections with respect to climate diversity. In general, considering climate and soil characteristics, specific adaptation patterns for each species group can be identified: Lycopersicon group (group 1) corresponds to species with lower altitude and higher mean annual temperature; species of Juglandifolia section (group 5) are those with the highest water availability, lowest pH, BD, and base saturation; species of Lycopersicoides section (group 6) are the ones with the highest altitude, the lowest mean annual temperature, and lowest water availability, groups 4 and 6 have the lowest water availability and soils with favorable agricultural characteristics, differing by altitude. The rest of the species (groups 2 and 3) are in the transition zones with the rest of the wild tomato species. One aspect to highlight is that when combining or considering climatic and edaphic information, it is possible to characterize in a better way the different groups, being able to better identify their differences and similarities.

Among possible uses of this approach is the identification of the germplasm with tolerance to adverse biotic and abiotic factors (Foolad and Lin., 2000; Mittova et al., 2004; Venema et al., 2005; Zhao et al., 2005; Ruiz-Corral et al., 2008; Chetelat et al., 2009; Arellano-Rodríguez et al., 2013; Ruiz-Corral et al., 2013; Cervantes-Moreno et al., 2014; Chen et al., 2015; Nosenko et al., 2016; Stam et al., 2017a; Stam et al., 2017b; Flores-Hernández et al., 2017; Razali et al., 2018; Dinh et al., 2019; Vilchez et al., 2019) with potential use for genetic breeding, identification of routes of germplasm accession, and areas of high and low diversity for use and conservation (Vilchez et al., 2019). In the information contained in Table 3, Table 4, and Figures 4, 5, it is possible to identify species with extreme values that indicate tolerance or resistance to climatic and edaphic factors, with potential use as germplasm for genetic breeding.

Finally, the hot spot analysis could satisfactorily identify regions with the greatest diversity of species. These are priority areas for conservation, either due to high or low diversity. Regions identified as of great importance for conservation comprise endemism. Diversity contained in populations with few isolated individuals or with restricted distribution could be more affected by environmental and anthropic changes. This result could be explained by the quantity and geographic distance between the accessions of species studied. However, this first approximation is very useful and agrees with the diversity results obtained for wild potato species in Peru (Hijmans and Spooner, 2001).

This research determines the most important edaphoclimatic descriptors of wild tomato species and its closely related species along their natural geographic range in South America. Patterns of climatic diversity correlate with species groups and sections proposed in current classification. New edaphic characteristics analyzed in the same areas were also useful, although with less discrimination than the climatic variables. Interaction between climatic and edaphic factors allows for understanding species distribution and their adaptation patterns. Another feature to highlight is the incorporation of new data from recent collections of specimens being properly identified (Ministerio del Ambiente, 2020) that were not considered before in other studies, and thus expanding precision and reliability of these results. Most important areas for conservation of wild tomato species and related outgroups were detected. Under this premise, this contribution is promissory for further ecogeographic study of wild tomatoes and closely related species at the local population scale, especially focused *in situ* conservation reserves as well as in localities outside protected areas. Edaphoclimatic descriptors in addition with other abiotic or biotic factors could help to better estimate the species ecological niches and determine local ecotypes. Selected descriptors would be tested in models of current and future distribution considering the impact of climate change and anthropic activities along the distribution range of these valuable genetic resources. Finally, this research can be used as a study model to replicate in other species.

## Data Availability

The data sets presented in this study can be found in online repositories. The names of the repository/repositories and accession number(s) can be found below: Solanacea source: http://solanaceaesource.org/; Tomato Genetic Resource Center: https://tgrc.ucdavis.edu; Global Biodiversity Information Facility: https://www.gbif.org.
