# Supplementary material for: Edaphoclimatic Descriptors of Wild Tomato Species (Solanum Sect. Lycopersicon) and Closely Related Species (Solanum Sect. Juglandifolia and Sect. Lycopersicoides) in South America
Source: Front Genet. 2021 Nov 17;12:748979. doi: 10.3389/fgene.2021.748979 (PMC8635747; doi:10.3389/fgene.2021.748979)
Supplement: Supplementary file 1 [file DataSheet2.PDF]

Table A1. Ecological descriptors for the rest of edaphoclimatic variables used in Canonical Correlation Analysis. Bio1=annual mean temperature, Bio2= mean diurnal range, Bio7= temperature annual range, Silt= silt percentage, CO= organic carbon, CEC= cation exchange capacity, Sodycity and Salinity. *Range (maximum-minimum value), **Median, ***(Coefficient of variation).

Group/Section	Species	Bio1 (°C)	Bio2 (°C)	Bio7 (°C)	Silt (%)	CO (%)	CEC (cmol/kg)	Sodycity (%)	Salinity (dS/m)
Lycopersicon	*S. cheesmaniae*	17.1-25.0*	7.6-10.3	12.1-14.1	29-49	0.3-2.9	7.0-34.0	1.0-4.0	0-0.4
		23.6** (3.2)***	9.0 (6.0)	13.0 (1.5)	30 (1.6)	1.3 (61.1)	33 (16.6)	1.0 (50)	0.1 (200)
	S. galapagense	20.1-25.0	7.6-10.1	12.2-14.1	29-49	0.3-29	7.0-34.0	1.0-4.0	0-0.4
		24.0 (2.9)	8.7 (5.5)	13 (3)	30 (0)	2.8 (28.3)	33.0 (16.6)	1.0 (50)	0 (0)
	S. pimpinellifolium	16.3-26.3	6.1-13.4	8.9-19.7	0-59	01-33.7	1.0-84.0	1.0-19	0-4.1
		22.6 (8.4)	9.9 (12.4)	15.1 (10.5)	30 (45)	0.5 (97.1)	10.0 (45)	2.0 (50)	0.1 (50)
Arcanum	*S. arcanum*	11.5-24.1	10.3-14.4	12.7-16.7	0-48	0.1-33.7	2.0-84.0	1.0-3.0	0-0.6
		18.1 (9.6)	13.0 (5.4)	15.0 (4.2)	30 (18.3)	2.0 (41.1)	14.0 (35.7)	1.0 (50)	0 (0)
	S. chmielewskii	12.8-20.5	11.8-15.7	15.4-21.2	23-57	0.4-2.3	4.0-22.0	1.0-13	0-1.2
		17.5 (9.5)	14.9 (3.1)	17.8 (3.0)	23 (8.6)	0.4 (25.6)	9.0 (8.3)	2 (0)	0.1 (0)
	S. neorickii	11.8-20.9	9.9-15.5	12.3-19.0	0-48	0.4-33.7	4.0-84.0	1.0-13.0	0-1.2
		17.1 (7.9)	12.6 (8.3)	14.5 (11.2)	19 (18.4)	2.3 (47.6)	11.0 (50)	1.0 (50)	0 (0)
Eriopersicon	*S. huaylasense*	11.3-20.3	10.8-14.0	13.4-16.2	23-48	0.3-3.5	9.0-24.0	1.0-3.0	0-0.4
		16.8 (9.6)	13.5 (3.7)	15.3 (3.8)	23 (54.3)	0.3 (285.9)	12.0 (50)	3 (33.3)	0 (0)
	S. corneliomulleri	9.6-18.3	9.4-16.6	14.3-20.6	16-48	0.3-5.5	6.0-27.0	1.0-3.0	0-0.6
		14.3 (11.9)	12.3 (5.8)	16.1 (4.6)	24 (14.5)	0.3 (265.6)	12.0 (25)	2.0 (25)	0 (0)
	S. peruvianum	9.6-20.9	4.7-15.5	11.6-19.5	4-55	0.1-3.5	1.0-25.0	1.0-65	0-56.1

		18.6 (7.1)	9.5 (18.5)	15.1 (5.2)	30 (11.6)	0.3 (44.9)	9.0 (16.6)	2.0 (25)	0.1 (150)
	S. chilense	5.4-20.4	4.9-18.6	11.8-27.4	3-55	0.2-5.5	3.0-27.0	1.0-65	0-56.1
		15.2 (19.1)	12.6 (19.0)	17.1 (13.7)	26 (21.1)	0.4 (37.7)	12.0 (35.4)	2.0 (25)	0.2 (75)
	S. habrochaites	7.0-25.8	6.5-15.0	10.6-19.3	0-59	0.1-33.7	1.0-84.0	1.0-4.0	0-0.6
		16.4 (16.9)	12 (6.6)	14.5 (8.2)	30 (18.3)	1.3 (60.5)	14.5 (27.5)	1.0 (100)	0 (0)
Neolycopersicon	*S. pennellii*	10.5-25.1	6.2-13.7	13.0-18.4	4-48	0.1-2.4	1.0-24.0	1.0-14.0	0-4.1
		18.4 (7.2)	10.3 (7.9)	15 (4.0)	30 (11.6)	0.3 (9.3)	9.0 (16.6)	2.0 (25)	0.2 (100)
Juglandifolia	*S. juglandifolium*	10.9-22.9	7.2-12.5	8.2-14.1	4-62	0.5-28	4.0-85.0	1.0-4.0	0-0.3
		15.9 (10.6)	9.1 (7.5)	10.3 (9.2)	37 (31)	5.3 (19.4)	21.0 (33.3)	1.0 (50)	0 (0)
	S. ochranthum	6.7-21.9	7.2-15.6	8.2-21.0	0-56	0.3-33.7	4.0-84.0	1.0-4.0	0-0.4
		14.0 (11.7)	11.5 (8.8)	13.3 (8.0)	30 (20.4)	1.8 (51.9)	14.0 (39.2)	1.0 (50)	0 (0)
Lycopersicoides	*S. lycopersicoides*	7.6-17.3	10.4-15.1	15.8-21.1	24-38	0.4-1.2	6.0-25.0	1.0-3.0	0-0.6
		11.3 (14.9)	14 (4.8)	19.0 (6.3)	29 (6.8)	0.7 (28.4)	13.0 (15.3)	2.0 (25)	0.4 (37.5)
	S. sitiens	8.4-13.2	15.6-17.6	21.8-25.6	19-29	0.4-0.5	6.0-16.0	1.0-3.0	0.1-0.4
		11.4 (7.6)	16.8 (0.9)	24.4 (1.6)	19 (15.7)	0.5 (7.7)	6.0 (58.3)	3.0 (0)	0.1 (50)

Table A2. Mean values of edaphoclimatic descriptors according climate types associated with 12 wild tomatoes (*Solanum* Sect. Lycopersicon) and 4 closely related species (*Solanum* Sect. Juglandifolia and Sect. Lycopersicoides). Alt (altitude, m), Bio1 (mean annual temperature, °C), Bio12 (annual precipitation, mm), ET (annual evapotranspiration, mm), pH (hydrogen ion concentration), CEC (cation exchange capacity, cmol/kg), BD (bulk density, kg/dm^3), SB (base solubility, %). Climate type: Af (tropical, rainforest), Am (tropical, monsoon), Aw (tropical, savannah), BWh (arid, desert, hot), BWk (arid, desert, cold), BSh (arid, steppe, hot), BSk (arid, steppe, cold), Csb (temperate, dry summer, warm summer), Cwb (temperate, dry winter, warm summer), Cfb (temperate, no dry season, warm summer), ET (polar, frost).

Section/Group	SPECIES	CLIMATE	ET	ALT	Bio12	Bio1	BD	pH	CEC	BS
Lycopersicon	*S. cheesmaniae*	**Af**	990	570	315	21.5	1.3	5.9	30.0	65.8
		**Aw**	494	179	289	23.3	1.3	6.3	30.7	73.1
		**BWh**	393	167	224	23.7	1.3	6.1	30.5	72.2
		**BSh**	516	102	380	23.8	1.3	6.7	25.8	73.4
		**Cfb**	1,018	1,084	319	19.3	1.2	4.7	28.8	31.4
	S. galapagense	**Aw**	551	207	292	23.3	1.3	6.2	28.9	65.7
		**BWh**	469	132	248	23.9	1.2	5.6	27.3	47.5
		**BSh**	457	34	275	24.4	1.2	5.1	29.2	39.4
	S. pimpinellifolium	**Af**	1,459	449	1,772	24.7	1.2	5.6	12.1	52.3
		**Am**	1,224	432	2,117	23.7	1.1	5.7	28.1	52.0
		**Aw**	878	123	1,313	24.9	1.3	6.1	19.4	79.9
		**BWh**	65	151	88	21.8	1.4	7.4	10.0	93.5
		**BWk**	89	1,288	115	17.4	1.1	6.7	34.0	90.8
		**BSh**	473	251	592	24.4	1.3	6.5	19.5	86.2
Arcanum	*S. arcanum*	**Am**	1,094	1,216	1,193	21.4	1.3	7.0	23.0	87.0
		**Aw**	564	1,411	705	20.4	1.1	5.5	19.9	53.6
		**BWh**	124	755	136	20.0	1.4	7.2	7.2	94.4
		**BWk**	177	1,818	228	17.4	1.4	5.7	10.0	62.5
		**BSh**	368	1,245	453	19.9	1.2	6.3	20.1	73.2
		**BSk**	354	2,431	393	15.5	1.2	6.1	21.1	78.1
		**Cwb**	619	2,461	710	15.9	1.3	6.4	16.7	82.8
		**Cfb**	714	2,135	849	16.7	1.3	6.0	17.1	74.8
	S. chmielewskii	**BSk**	545	2,349	703	17.8	1.3	7.0	10.8	78.0
		**Cwb**	636	2,512	952	16.9	1.2	7.8	11.3	96.7
	S. neorickii	**Aw**	660	1,458	759	20.1	0.3	4.9	84.0	45.0
		**BSk**	483	2,106	713	18.5	1.3	5.8	11.6	59.8
		**Cwb**	616	2,482	918	16.9	1.2	6.6	14.6	74.6
		**Cfb**	716	2,215	838	16.5	1.0	5.7	26.7	58.1
Eriopersicon	*S. huaylasense*	**BWh**	167	1,307	228	19.6	1.3	6.8	19.1	84.5
		**BSh**	213	1,418	291	19.6	1.3	6.5	19.5	76.8
		**BSk**	283	2,471	346	15.9	1.3	6.2	17.2	64.0
	S. corneliomulleri	**BWk**	100	2,019	137	15.3	1.3	6.2	12.0	61.2

Section/Group	SPECIES	CLIMATE	ET	ALT	Bio12	Bio1	BD	pH	CEC	BS
		**BSk**	243	2,645	359	11.9	1.4	5.7	13.6	39.7
	S. peruvianum	**BWh**	13	346	25	19.2	1.4	7.6	8.4	94.2
		**BWk**	61	1,649	81	16.1	1.4	6.6	11.1	70.1
		**BSk**	250	2,532	368	12.9	1.3	6.1	17.0	57.8
	S. chilense	**BWh**	8	561	9	18.9	1.4	7.9	8.2	99.0
		**BWk**	41	1,962	48	13.9	1.3	7.3	11.3	92.9
		**BSk**	214	3,499	256	8.8	1.4	6.4	11.7	72.8
	S. habrochaites	**Af**	1,484	677	1,683	24.2	1.1	5.7	15.4	40.2
		**Aw**	823	1,285	945	20.9	1.2	6.2	17.0	69.4
		**BWh**	108	515	132	21.1	1.4	7.5	9.5	94.4
		**BWk**	136	1,776	191	15.8	1.4	6.1	13.4	58.0
		**BSh**	480	1,026	557	21.1	1.3	7.0	15.2	86.0
		**BSk**	346	2,612	437	13.8	1.3	6.1	17.0	66.5
		**Csb**	581	2,692	608	13.8	1.3	6.3	10.4	85.9
		**Cwb**	648	2,705	741	14.5	1.3	6.6	18.0	78.9
		**Cwc**	556	3,470	727	9.2	1.3	6.5	22.0	89.0
		**Cfb**	790	2,293	944	15.9	1.2	5.9	20.7	61.2
		**ET**	556	3,514	619	9.1	1.4	5.8	13.2	50.2
Neolycopersicon	*S. pennellii*	**BWh**	33	587	47	19.6	1.4	7.6	8.7	93.7
		**BWk**	79	1,533	105	16.5	1.4	6.4	11.7	60.2
		**BSk**	244	2,586	336	13.0	1.4	5.9	13.5	47.0
Juglandifolia	*S. juglandifolium*	**Af**	1,361	1,475	2,227	20.0	0.7	5.0	45.4	26.4
		**Am**	1,208	1,324	2,119	19.6	0.8	5.3	27.3	18.8
		**Aw**	840	1,537	998	18.3	1.2	5.6	16.8	69.8
		**Csb**	1,086	2,101	1,472	15.8	0.9	5.2	16.5	17.4
		**Cwb**	782	2,580	822	13.3	1.0	5.3	15.5	25.9
		**Cfb**	1,153	2,301	1,813	15.2	0.9	5.2	27.1	26.7
		**ET**	1,015	3,144	1,222	10.9	1.4	6.2	7.0	86.0
	S. ochranthum	**Af**	1,271	1,453	1,961	20.1	0.8	5.6	44.0	46.0
		**Aw**	754	1,749	838	19.3	1.2	7.9	22.3	100.0
		**BSk**	502	2,611	646	15.2	1.2	7.0	12.1	86.7
		**Csb**	973	2,574	1,074	14.3	1.1	5.8	12.5	48.0
		**Cwb**	709	3,047	976	13.7	1.3	7.3	10.2	87.3
		**Cfb**	847	2,581	1,094	14.3	1.1	5.5	18.7	50.5
		**ET**	833	3,479	1,132	9.0	1.2	5.9	13.3	56.4
Lycopersicoides	*S. lycopersicoides*	**BWk**	83	2,842	103	11.8	1.3	7.1	13.0	88.8
		**BSk**	181	3,653	213	8.2	1.4	6.5	12.0	87.0
	S. sitiens	**BWk**	20	2,773	17	11.3	1.3	7.1	9.2	93.2

Table A3. Mean values of edaphoclimatic descriptors according soil types associated with 12 wild tomatoes (*Solanum* Sect. Lycopersicon) and 4 closely related species (*Solanum* Sect. Juglandifolia and Sect. Lycopersicoides). Alt (altitude, m), Bio1 (mean annual temperature, °C), Bio12 (annual precipitation, mm), ET (annual evapotranspiration, mm), pH (hydrogen ion concentration), CEC (cation exchange capacity, cmol/kg), BD (bulk density, kg/dm^3), SB (base solubility, %). Soil type: AC (acrisol), AN (andosol), AR (arenosol), CH (chernozem), CM (cambisol), FL (fluvisol), GL (gleysol), HS (histosol), KS (kastanozem), LP (leptosol), LV (luvisol), PH (phaeozem), PL (planosol), RG (regosol), SC (solonchak), SN (solonetz), VR (vertisol).

Section/Group	SPECIES	SOIL	ET	Alt	Bio12	Bio1	BD	pH	CEC	BS
Lycopersicon	*S. cheesmaniae*	**CM**	576	357	300	22.5	1.3	4.9	34.0	31.0
		**FL**	698	409	262	22.7	1.1	4.3	20.0	32.0
		**LP**	428	141	248	23.5	1.3	7.1	33.0	100.0
		**PH**	510	88	546	23.4	1.4	6.2	7.0	86.0
		**RG**	499	87	408	23.8	1.3	8.5	23.0	100.0
	S. galapagense	**CM**	456	101	285	23.8	1.3	4.9	34.0	31.0
		**FL**	602	150	267	24.0	1.1	4.3	20.0	32.0
		**LP**	583	259	326	22.9	1.3	7.1	33.0	100.0
		**PH**	504	88	546	23.4	1.4	6.2	7.0	86.0
		**RG**	501	202	194	23.5	1.3	8.5	23.0	100.0
	S. pimpinellifolium	**AC**	1,684	151	2,436	26.2	1.4	4.9	7.0	43.0
		**AN**	1,170	231	2,393	24.1	0.9	5.8	28.0	39.4
		**AR**	55	101	78	21.5	1.5	8.1	2.8	98.9
		**CH**	87	490	117	20.7	1.2	7.0	24.0	100.0
		**CM**	372	139	532	24.3	1.4	7.2	15.6	96.4
		**FL**	112	107	164	21.9	1.3	7.2	11.5	92.4
		**GL**	90	58	110	24.0	1.3	5.2	18.0	21.0
		**HS**	264	1,091	296	20.1	0.3	4.9	84.0	45.0
		**KS**	208	67	244	25.6	1.3	7.4	25.0	99.0
		**LP**	121	225	156	21.9	1.3	7.2	10.8	89.9
		**LV**	829	144	1,150	24.8	1.4	5.9	22.1	86.9
		**PH**	224	157	274	22.3	1.3	6.3	22.4	85.9
		**PL**	280	96	403	24.4	1.5	5.9	7.0	75.0
		**RG**	356	325	417	21.9	1.3	5.8	13.2	54.6
		**SC**	7	377	1	19.2	1.3	8.0	5.0	100.0
		**SN**	6	17	17	19.2	1.3	7.4	18.0	96.0
Arcanum	*S. arcanum*	**AR**	50	611	49	19.7	1.5	8.5	2.0	100.0
		**CH**	409	2,139	492	17.1	1.2	7.0	24.0	100.0
		**CM**	677	2,323	774	16.1	1.3	5.7	19.6	89.6
		**GL**	327	2,962	345	14.2	1.3	5.2	18.0	21.0
		**HS**	348	1,943	391	17.0	0.3	4.9	84.0	45.0
		**KS**	596	2,551	698	15.3	1.3	7.4	25.0	99.0
		**LP**	397	1,634	461	18.2	1.3	6.0	10.4	78.9

Section/Group	SPECIES	SOIL	ET	Alt	Bio12	Bio1	BD	pH	CEC	BS
		**PH**	637	1,754	728	18.1	1.3	6.5	22.0	89.0
		**RG**	517	1,886	614	17.7	1.3	7.1	16.6	74.8
	S. chmielewskii	**LP**	545	2,381	1,006	18.5	1.3	7.3	15.0	93.0
		**PH**	552	2,213	913	18.6	1.3	6.5	22.0	89.0
		**RG**	654	2,583	930	16.5	1.2	8.0	8.8	96.4
		**SN**	586	2,253	991	18.2	1.5	7.4	15.0	100.0
	S. neorcikii	**CH**	575	2,090	763	17.1	1.2	7.0	24.0	100.0
		**CM**	704	2,305	847	16.0	1.3	5.7	19.1	82.3
		**HS**	666	1,908	787	18.1	0.3	4.9	84.0	45.0
		**LP**	624	2,381	821	16.5	1.1	5.1	11.4	38.3
		**LV**	783	2,232	914	16.1	1.5	6.0	20.0	75.0
		**PH**	572	2,203	1,042	18.9	1.3	6.5	22.0	89.0
		**RG**	687	2,312	894	17.2	1.2	7.5	13.5	89.6
		**SN**	631	2,018	1,126	19.8	1.5	7.4	15.0	100.0
		**VR**	702	2,425	804	15.1	1.7	7.9	39.0	100.0
Eriopersicon	*S. huaylasense*	**CH**	240	2,272	290	16.5	1.2	7.0	24.0	100.0
		**GL**	329	2,684	399	15.6	1.3	5.2	18.0	21.0
		**LP**	285	2,229	363	16.7	1.4	5.7	11.9	39.4
	S. corneliomulleri	**CH**	151	2,128	235	14.5	1.2	7.0	24.0	100.0
		**KS**	53	2,220	64	15.8	1.3	7.4	25.0	99.0
		**LP**	156	2,056	228	14.4	1.4	6.3	11.9	57.1
		**PL**	81	2,567	89	14.2	1.4	4.7	6.0	34.0
		**RG**	197	2,542	279	12.7	1.2	5.8	14.1	37.0
	S. peruvianum	**AR**	20	230	44	19.0	1.5	7.9	4.7	97.6
		**CH**	196	1,907	256	14.9	1.2	7.0	24.0	100.0
		**FL**	12	285	21	18.8	1.4	7.4	11.5	97.1
		**GL**	5	278	11	19.7	1.3	5.2	18.0	21.0
		**KS**	13	1,197	14	17.5	1.3	7.4	25.0	99.0
		**LP**	47	1,035	71	17.8	1.3	7.3	10.1	85.0
		**PL**	68	2,435	67	15.0	1.4	4.7	6.0	34.0
		**RG**	65	1,300	95	16.5	1.3	5.8	10.9	51.9
		**SC**	6	482	9	18.9	1.3	8.1	6.4	100.0
	S. chilense	**AN**	42	3,918	54	6.5	1.0	5.8	17.0	45.0
		**AR**	13	259	17	17.3	1.5	8.3	3.3	99.7
		**FL**	8	772	7	18.2	1.3	7.1	13.3	93.4
		**GL**	6	425	2	17.1	1.3	6.1	13.0	81.0
		**KS**	39	2,075	46	15.4	1.3	7.4	25.0	99.0
		**LP**	48	1,996	56	13.8	1.3	7.3	13.6	94.6
		**LV**	236	3,995	259	5.7	1.5	7.7	10.0	100.0
		**PL**	115	3,169	138	11.4	1.4	4.7	6.0	34.0
		**RG**	32	2,410	36	13.7	1.2	7.2	8.7	93.4
		**SC**	8	793	6	17.9	1.4	8.2	7.1	100.0

Section/Group	SPECIES	SOIL	ET	Alt	Bio12	Bio1	BD	pH	CEC	BS
	S. habrochaites	**AN**	1,149	1,797	1,368	17.9	1.0	5.3	14.3	19.5
		**AR**	118	246	169	21.8	1.5	8.2	2.3	99.8
		**CH**	354	2,465	448	14.3	1.2	7.0	24.0	100.0
		**CM**	686	1,675	797	18.7	1.4	6.4	15.2	74.4
		**FL**	373	256	490	21.8	1.3	6.2	14.3	70.9
		**GL**	320	2,888	362	14.2	1.3	5.2	18.0	21.0
		**HS**	537	2,012	654	16.9	0.3	4.9	84.0	45.0
		**KS**	643	2,697	735	14.7	1.3	7.4	25.0	99.0
		**LP**	400	2,087	478	15.9	1.3	5.8	11.0	55.2
		**LV**	881	1,710	1,014	17.7	1.3	5.7	22.0	97.0
		**PH**	584	2,421	674	15.0	1.3	6.4	15.8	87.3
		**PL**	458	47	563	25.6	1.5	5.9	7.0	75.0
		**RG**	638	2,203	741	16.3	1.2	6.9	18.1	73.1
		**VR**	563	2,142	635	16.3	1.7	7.9	39.0	100.0
Neolycopersicon	*S. pennellii*	**AR**	16	339	24	20.3	1.5	7.6	5.9	96.4
		**CH**	51	1,118	64	17.7	1.2	7.0	24.0	100.0
		**FL**	41	214	58	20.4	1.4	7.6	10.0	100.0
		**LP**	59	1,068	81	18.1	1.4	7.0	10.2	77.6
		**RG**	83	1,651	113	16.7	1.3	7.0	12.8	69.6
		**SC**	5	381	1	19.4	1.3	8.0	5.0	100.0
Juglandifolia	*S. juglandifolium*	**AN**	1,151	2,097	1,768	16.0	0.8	5.2	28.8	18.5
		**AR**	861	2,516	1,080	13.6	1.9	5.4	4.0	70.0
		**CM**	1,172	2,111	1,830	16.4	1.2	4.9	27.8	32.9
		**LP**	1,181	2,235	1,906	15.7	0.9	5.1	26.4	26.4
		**LV**	823	1,931	967	16.6	1.3	5.7	21.7	93.9
		**PH**	843	2,843	972	13.0	1.4	6.2	7.0	86.0
		**RG**	860	2,405	1,028	15.1	1.2	7.7	22.0	100.0
	S. ochranthum	**AN**	943	2,688	1,173	13.4	1.0	5.7	20.0	37.8
		**CH**	739	2,401	952	15.1	1.2	7.0	24.0	100.0
		**CM**	807	2,572	1,057	14.3	1.3	5.5	17.3	62.4
		**HS**	677	2,118	883	16.9	0.3	4.9	84.0	45.0
		**LP**	837	2,614	1,159	14.2	1.1	5.2	15.7	39.8
		**PH**	911	3,043	1,065	11.7	1.4	6.2	7.0	86.0
		**RG**	720	2,880	914	14.2	1.2	7.5	12.8	90.0
		**VR**	562	2,453	566	14.3	1.7	7.9	39.0	100.0
Lycopersicoides	*S. lycopersicoides*	**KS**	82	2,960	107	11.9	1.3	7.4	25.0	99.0
		**LP**	89	2,894	109	11.4	1.3	7.4	13.7	95.9
		**PL**	101	3,002	126	12.2	1.4	4.7	6.0	34.0
		**RG**	16	1,932	25	14.9	1.4	7.9	13.0	100.0
	S. sitiens	**LP**	24	3,163	27	9.2	1.3	7.5	16.0	100.0
		**RG**	20	2,744	17	11.4	1.2	7.0	8.7	92.7
