# Supplementary material for: Edaphoclimatic Descriptors of Wild Tomato Species (Solanum Sect. Lycopersicon) and Closely Related Species (Solanum Sect. Juglandifolia and Sect. Lycopersicoides) in South America
Source: Front Genet. 2021 Nov 17;12:748979. doi: 10.3389/fgene.2021.748979 (PMC8635747; doi:10.3389/fgene.2021.748979)

Figure S1. Geographic distribution of wild tomatoes and closely related species.

Solanum Sect. Lycopersicon, Lycopersicon group

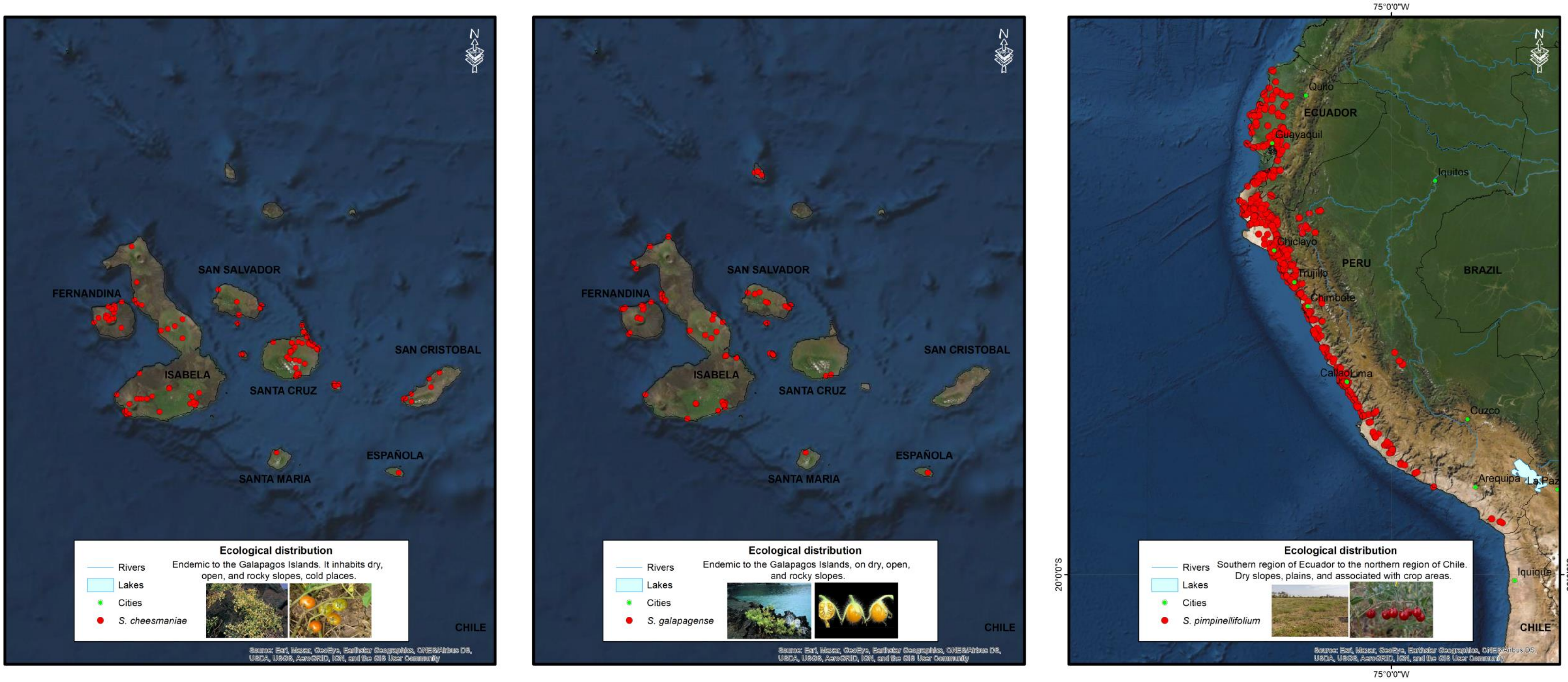

Solanum Sect. Lycopersicon, Arcanum group

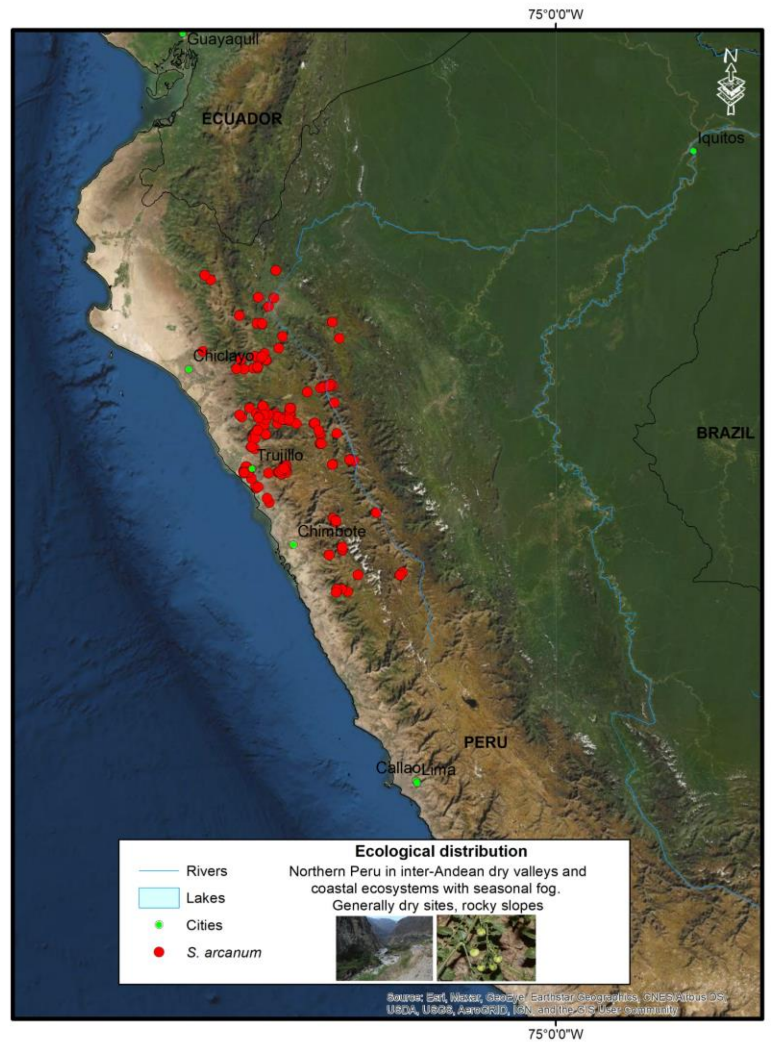

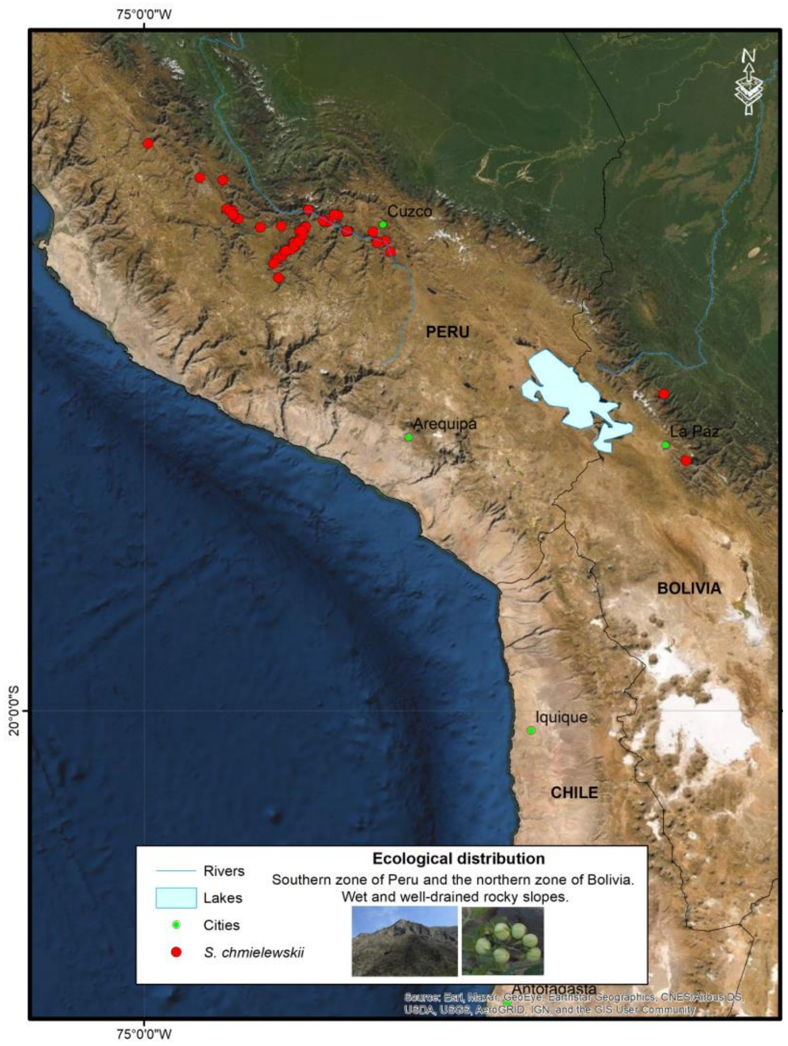

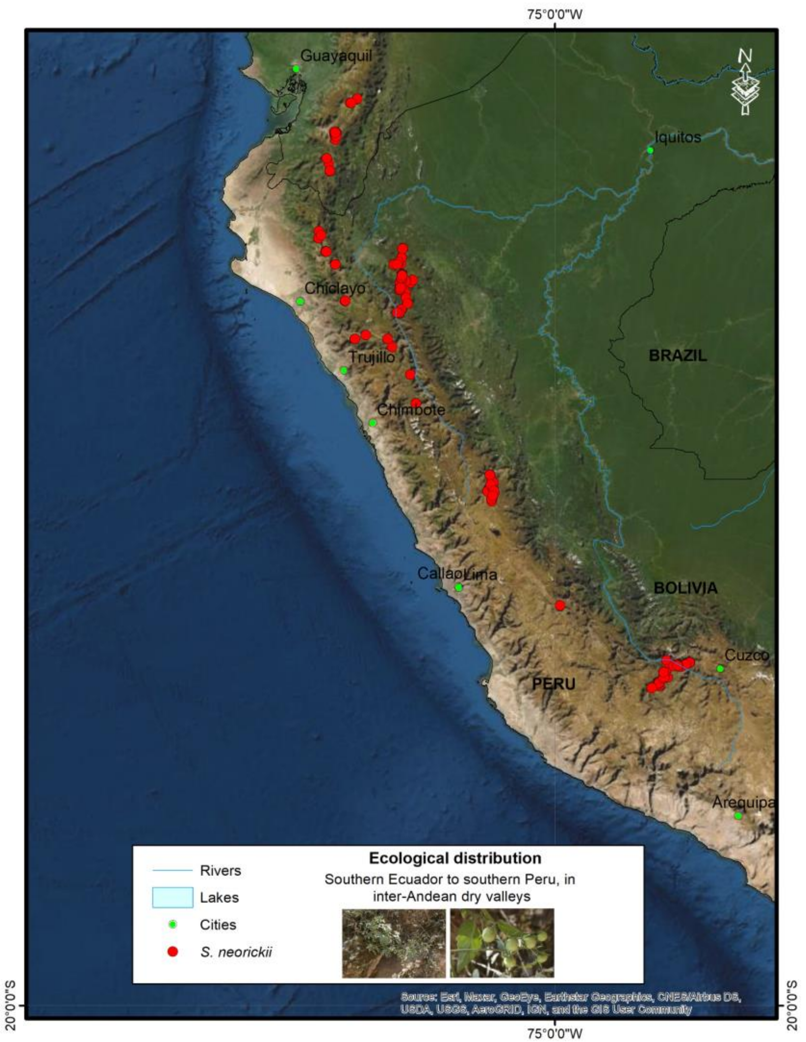

Solanum Sect. Lycopersicon, Eriopersicon group

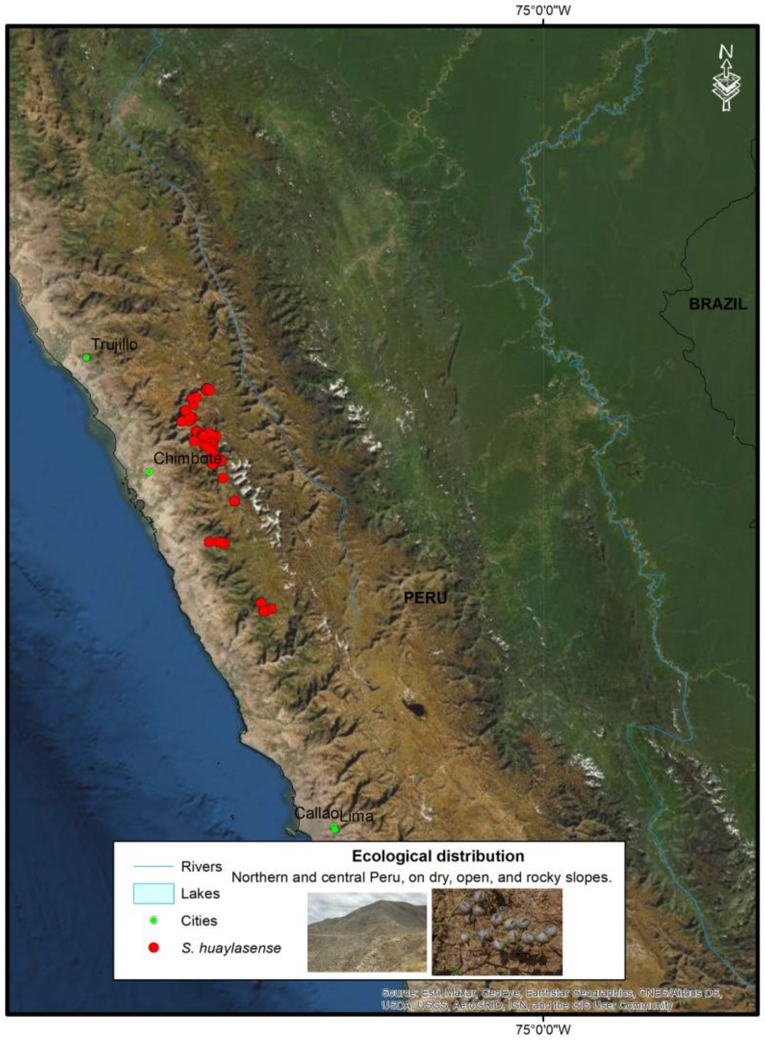

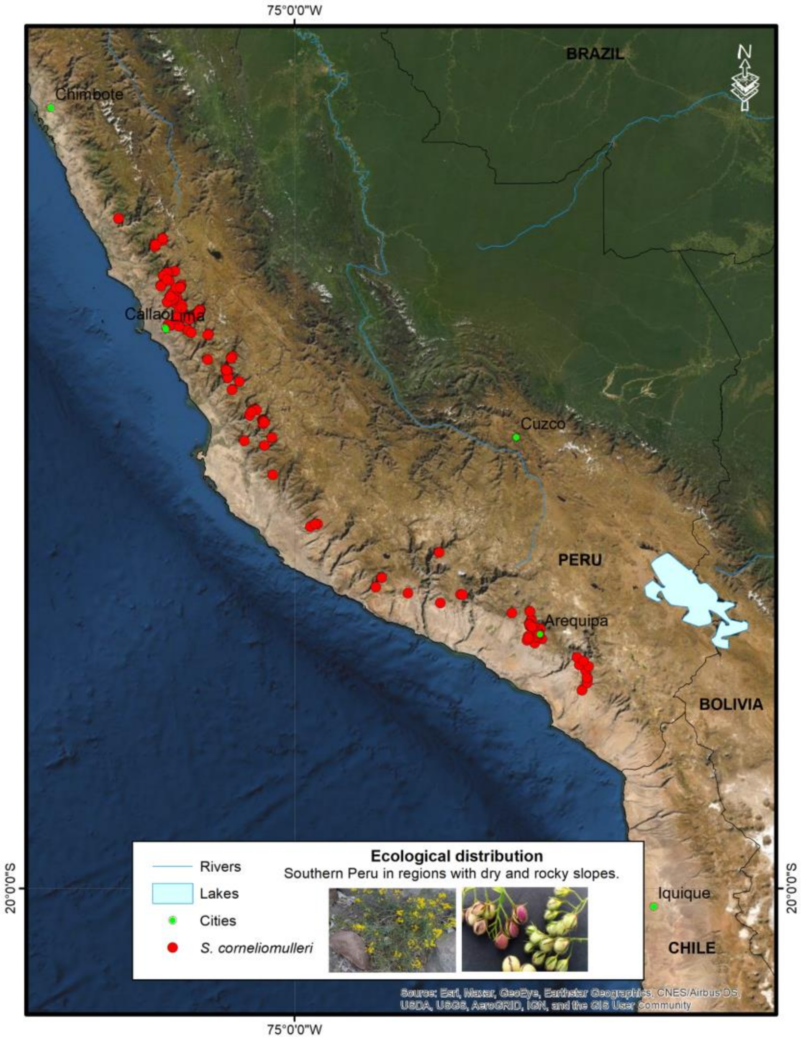

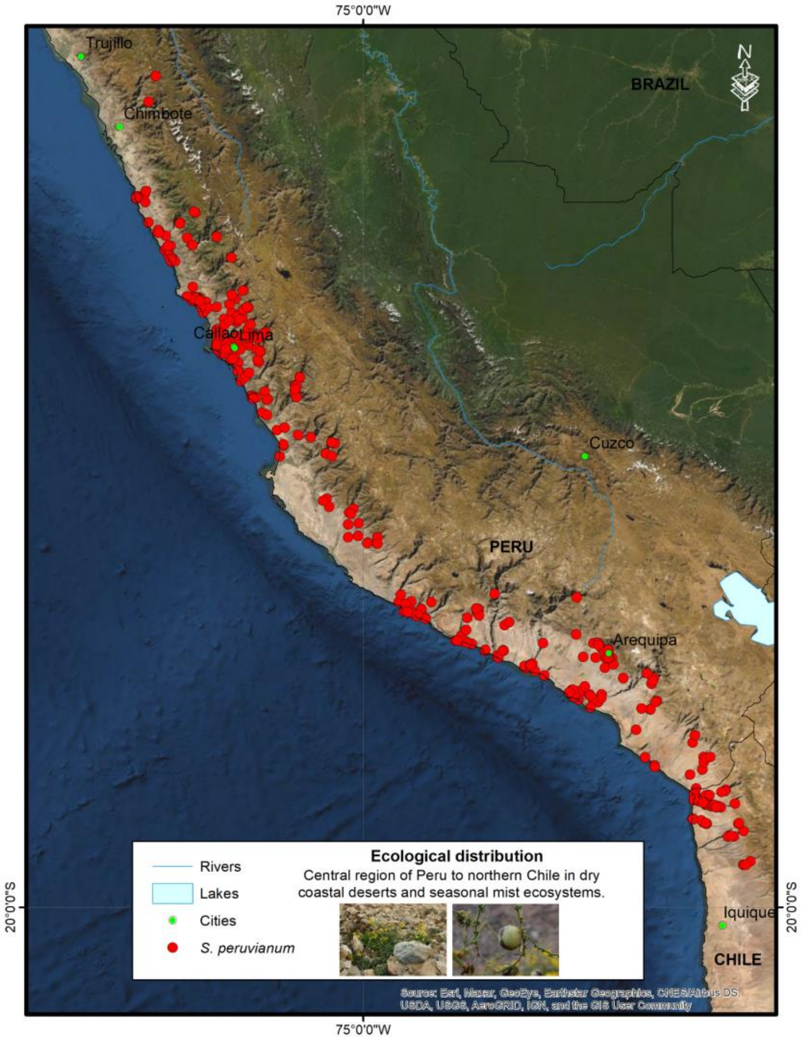

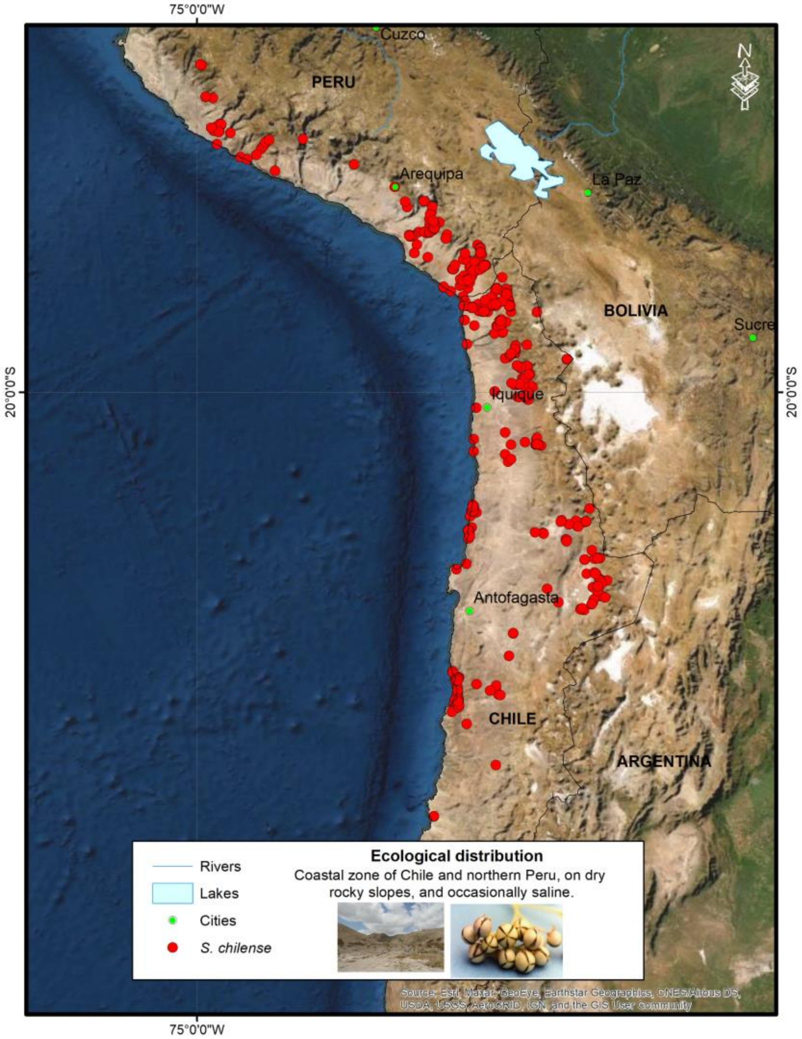
75°0'0"W
20°0'0"S
Cuzco
PERU
Arequipa
La Paz
BOLIVIA
Sucre
Iquique
Antofagasta
CHILE
ARGENTINA
N
Ecological distribution
Coastal zone of Chile and northern Peru, on dry
rocky slopes, and occasionally saline.
Rivers
Lakes
Cities
S. chilense
Source: Esri, Maxar, GeoEye, Earthstar Geographics, CNES/Airbus DS,
USDA, USGS, AeroGRID, IGN, and the GIS User Community

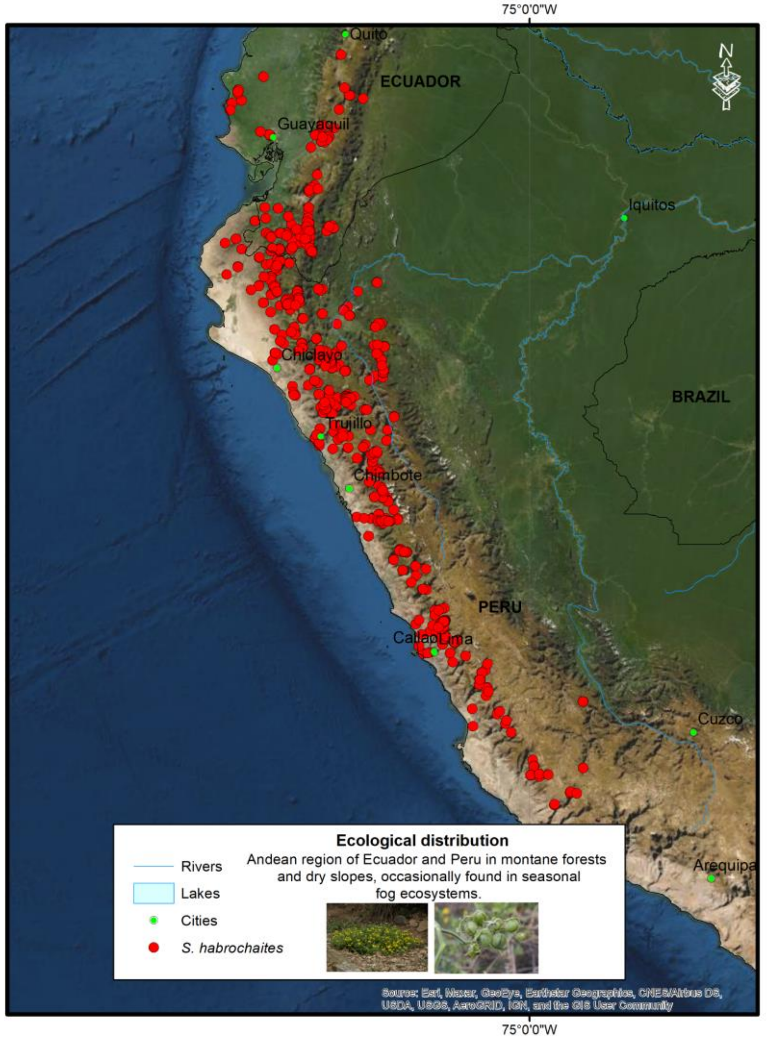
75°0'0"W
Quito
ECUADOR
Guayaquil
Iquitos
BRAZIL
Chiclayo
Trujillo
Chimbote
PERU
Callao
Lima
Cuzco
Arequipa
N
Ecological distribution
Andean region of Ecuador and Peru in montane forests
and dry slopes, occasionally found in seasonal
fog ecosystems.
Rivers
Lakes
Cities
S. habrochaites
Source: Esri, Maxar, GeoEye, Earthstar Geographics, CNES/Airbus DS,
USDA, USGS, AeroGRID, IGN, and the GIS User Community

Solanum Sect. Lycopersicon, Neolypersicon group

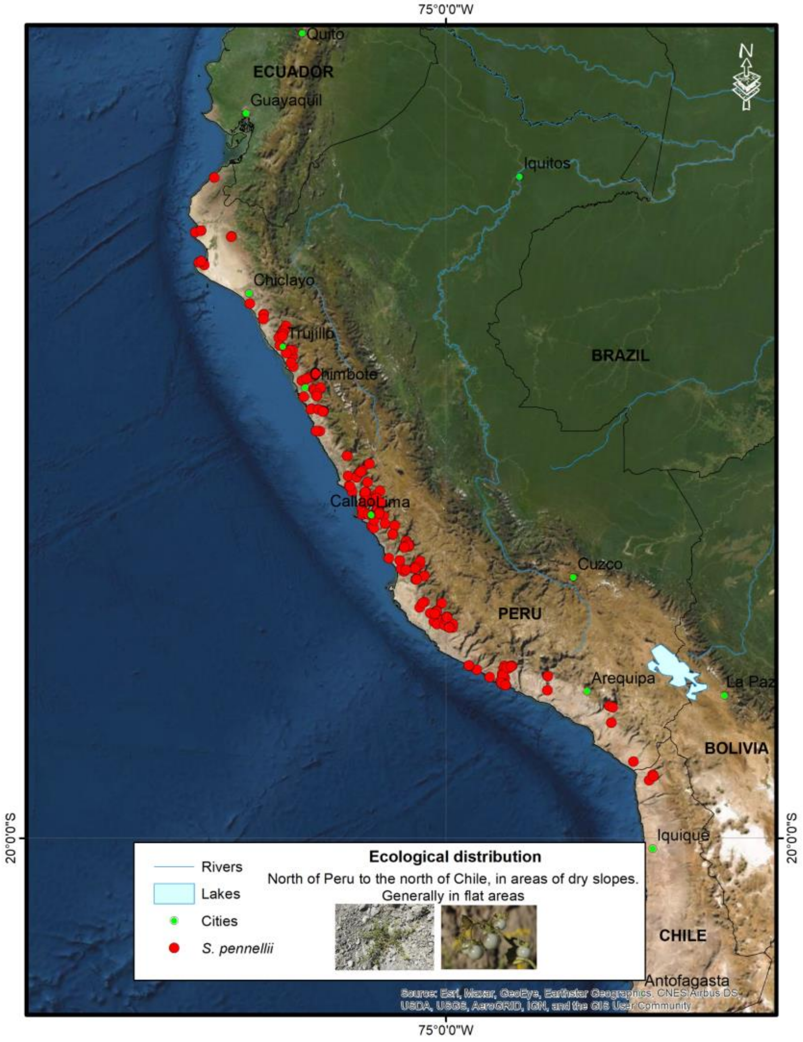

Solanum Sect. Juglandifolia

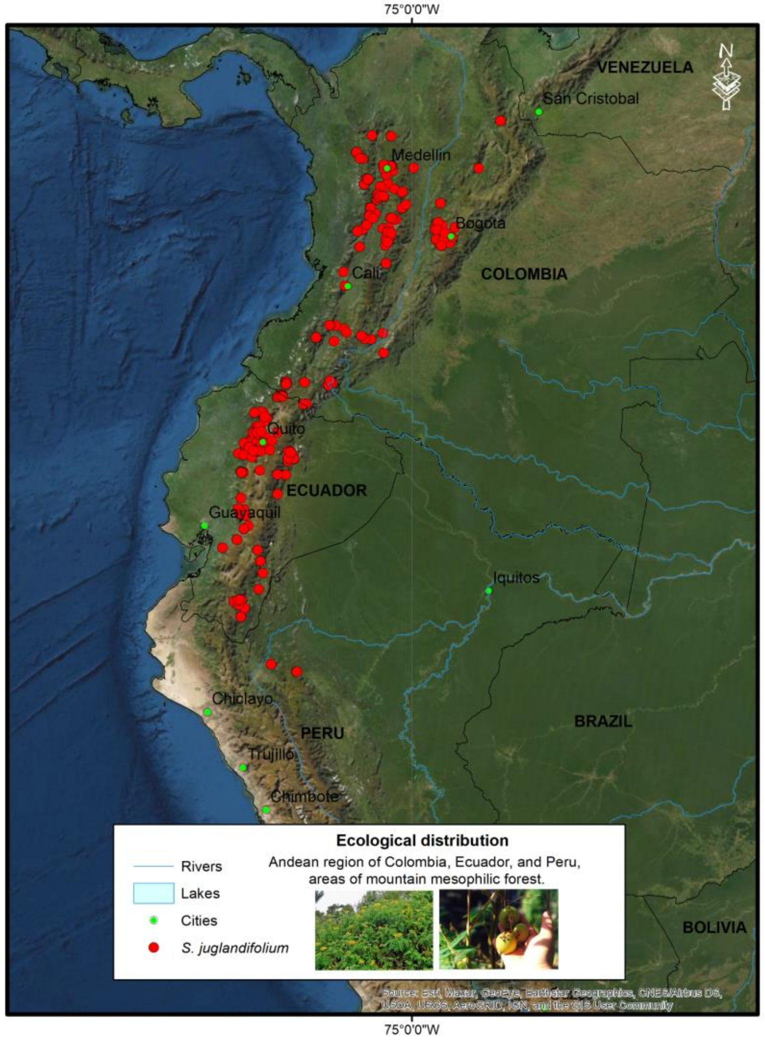

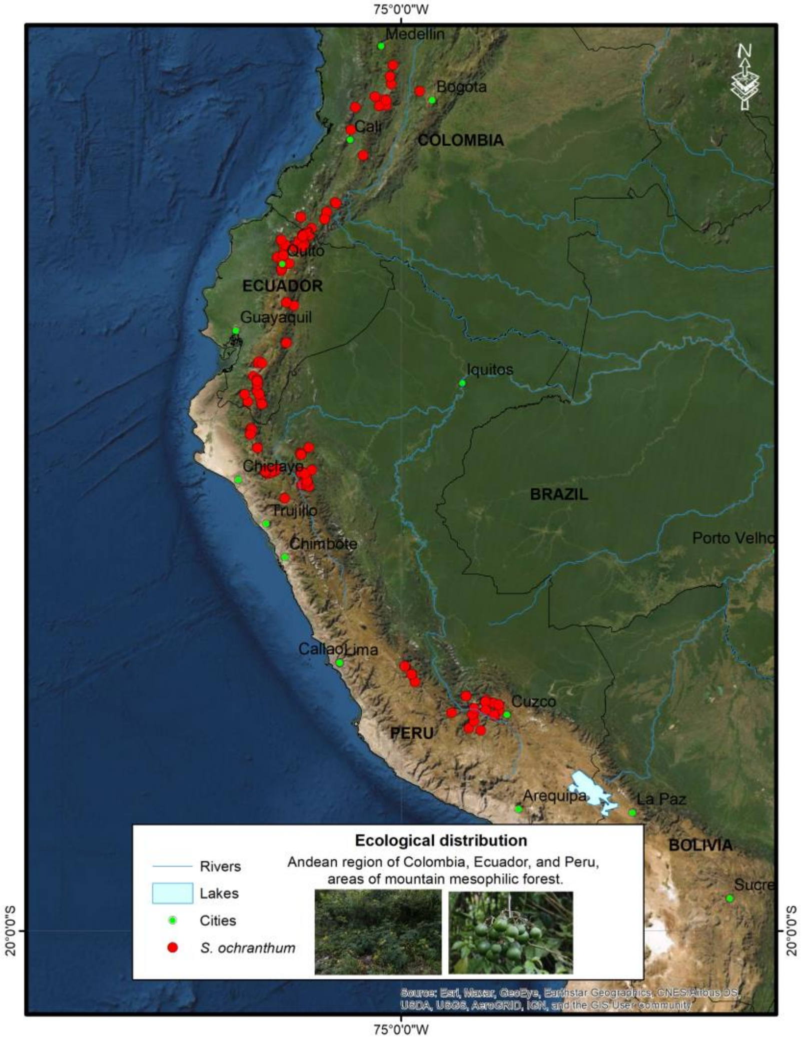

Solanum Sect. Lycopersicoides

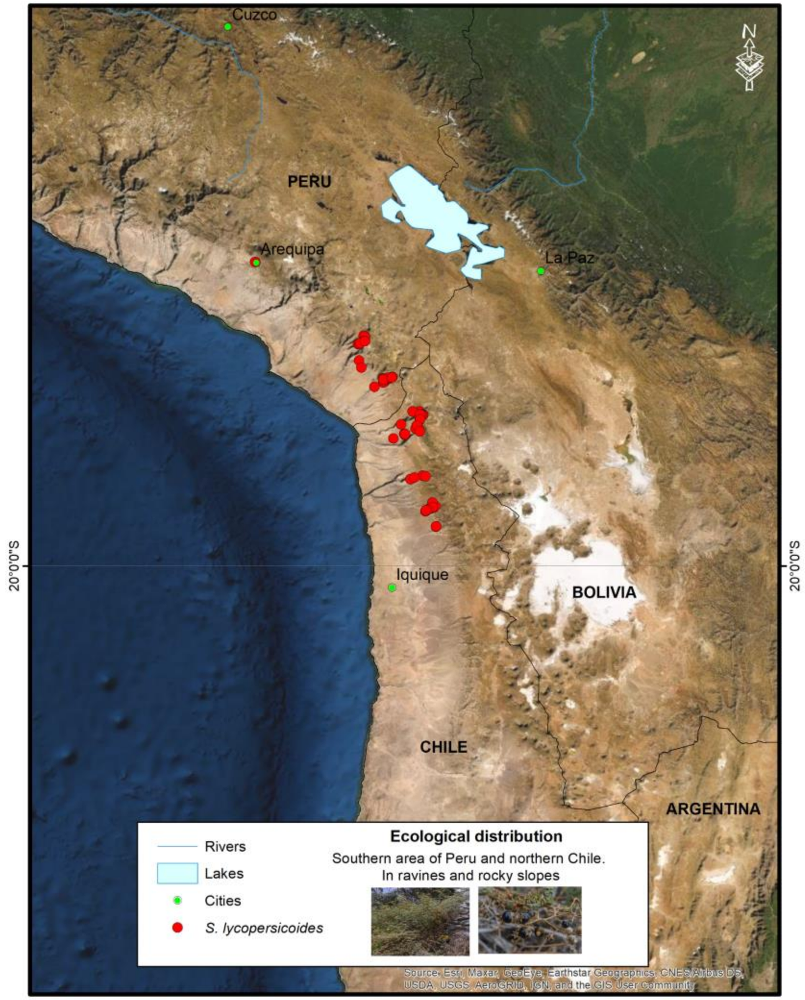

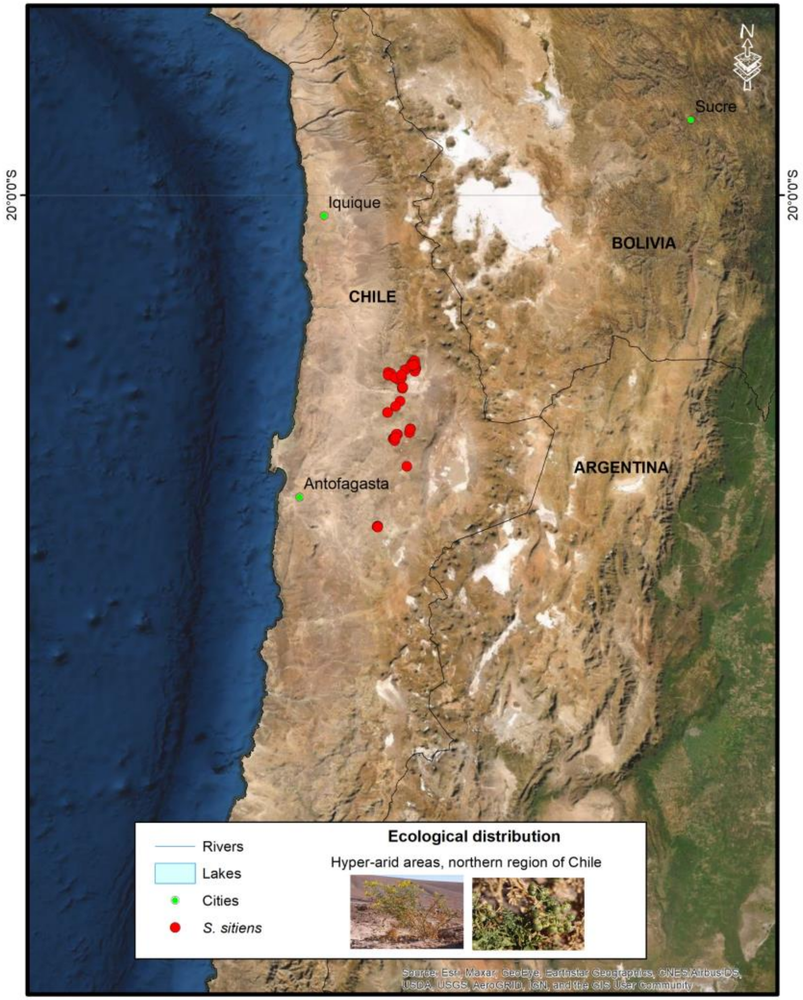

Supplement: Supplementary file 2 [file DataSheet1.PDF]
